# Circadian clock component REV-ERB**α** controls homeostatic regulation of pulmonary inflammation

**DOI:** 10.1172/JCI93910

**Published:** 2018-04-30

**Authors:** Marie Pariollaud, Julie E. Gibbs, Thomas W. Hopwood, Sheila Brown, Nicola Begley, Ryan Vonslow, Toryn Poolman, Baoqiang Guo, Ben Saer, D. Heulyn Jones, James P. Tellam, Stefano Bresciani, Nicholas C.O. Tomkinson, Justyna Wojno-Picon, Anthony W.J. Cooper, Dion A. Daniels, Ryan P. Trump, Daniel Grant, William Zuercher, Timothy M. Willson, Andrew S. MacDonald, Brian Bolognese, Patricia L. Podolin, Yolanda Sanchez, Andrew S.I. Loudon, David W. Ray

**Affiliations:** 1 Faculty of Biology, Medicine and Health, University of Manchester, Manchester, United Kingdom.; 2 Department of Pure and Applied Chemistry, University of Strathclyde, Glasgow, United Kingdom.; 3 GlaxoSmithKline R&D, Stevenage, United Kingdom.; 4 Molecular Discovery Research, GlaxoSmithKline, Research Triangle Park, North Carolina, USA.; 5 Novartis AG, East Hannover, New Jersey, USA.; 6 Eshelman School of Pharmacy, University of North Carolina at Chapel Hill, Chapel Hill, North Carolina, USA.; 7 Stress and Repair Discovery Performance Unit, Respiratory Therapy Area, GlaxoSmithKline, King of Prussia, Pennsylvania, USA.

**Keywords:** Inflammation, Pulmonology, Innate immunity, Mouse models, Neutrophils

## Abstract

Recent studies reveal that airway epithelial cells are critical pulmonary circadian pacemaker cells, mediating rhythmic inflammatory responses. Using mouse models, we now identify the rhythmic circadian repressor REV-ERBα as essential to the mechanism coupling the pulmonary clock to innate immunity, involving both myeloid and bronchial epithelial cells in temporal gating and determining amplitude of response to inhaled endotoxin. Dual mutation of REV-ERBα and its paralog REV-ERBβ in bronchial epithelia further augmented inflammatory responses and chemokine activation, but also initiated a basal inflammatory state, revealing a critical homeostatic role for REV-ERB proteins in the suppression of the endogenous proinflammatory mechanism in unchallenged cells. However, REV-ERBα plays the dominant role, as deletion of REV-ERBβ alone had no impact on inflammatory responses. In turn, inflammatory challenges cause striking changes in stability and degradation of REV-ERBα protein, driven by SUMOylation and ubiquitination. We developed a novel selective oxazole-based inverse agonist of REV-ERB, which protects REV-ERBα protein from degradation, and used this to reveal how proinflammatory cytokines trigger rapid degradation of REV-ERBα in the elaboration of an inflammatory response. Thus, dynamic changes in stability of REV-ERBα protein couple the core clock to innate immunity.

## Introduction

Protection of pulmonary mucosal surfaces from infection requires complex interplay between the airway epithelial cells and mononuclear phagocyte populations both resident within the lung (1). Airway macrophages function as required accessory cells for optimal lung performance, with adverse consequences arising from either insufficient or excessive activation. Bronchial epithelial cells maintain an intact mucosal barrier, and transmit inhibitory signals to airway macrophages, maintaining homeostatic control of airway inflammation. In response to bacterial infection, neutrophil recruitment into the inflamed lung from the circulating neutrophil compartment is a necessary and closely regulated phenomenon, with avoidable tissue damage the consequence for excessive neutrophil infiltration. The determinants and controls maintaining pulmonary immune homeostasis and regulating inflammatory responses remain poorly defined. Recently, we discovered that the circadian clock exerted a major impact on the airway inflammatory response to challenge (2).

The central clock lies in the suprachiasmatic nucleus of the brain and maintains essential synchrony of peripheral tissue clocks via neural and humoral mediators. Virtually all cells in the body express components of the cellular circadian clock and are capable of sustaining circadian oscillations. This cellular circadian oscillator consists of a positive arm — CLOCK and BMAL1 heterodimers — driving transcription of 2 inhibitory arms — PER/CRY and REV-ERBα/REV-ERBβ (also known as NR1D1 and NR1D2), which feed back to inhibit BMAL1/CLOCK heterodimer transactivation function (3). The circadian clock powerfully regulates inflammation (4–6). Multiple measures of innate immunity show strong time-of-day variation, including cell trafficking to tissues, and monocyte/macrophage responses to TLR activation (7, 8), but far less is known about circadian control of inflammation in an organ context, such as the lung. Previously, we identified a strong time-of-day variation in pulmonary inflammation, and response to pneumococcal infection, a prevalent infectious challenge to the lung (2). This study showed that circadian clock disruption by deletion of the essential clock gene BMAL1 in the myelomonocytic cells had little impact, but that BMAL1 deletion in the airway epithelial cells, driven by CCSP-iCre transgene, augmented inflammation across the circadian cycle. The circadian effect in epithelial cells was explained by aberrant regulation of a single dominant neutrophil chemokine, CXCL5.

BMAL1 plays many roles in addition to being a core circadian clock component. The immune regulatory functions of BMAL1 have been proposed to lie with regulation of the downstream REV-ERB transcription factors (5), CLOCK regulation of NF-ĸB function (9), and microRNA regulation (10). BMAL1 is not tractable to drug therapy; therefore, identifying how BMAL1 regulates lung inflammation is important to realize benefits in the clinic. The orphan nuclear receptor REV-ERBα has been shown to be an important intermediary molecular link between the core clock and inflammatory pathways in macrophages (5), possibly mediated by direct DNA binding to the promoters of proinflammatory chemokines (11), but also involving inhibition of distal proinflammatory cytokine enhancers selected by macrophage-lineage-determining factors (12).

New insights into REV-ERB biology have resulted from loss-of-function studies, and application of chemical biology tools to manipulate function (5, 13–15). These include the discovery that REV-ERBα and β are functionally redundant in terms of maintaining circadian oscillations, with only a minor change in period length seen in REV-ERBα–null mice (13, 16). However, a fascinating discovery was the functional dissociation between the DNA-binding domain (DBD) functions of REV-ERBα, required for circadian clock regulation, and the DBD-independent functions, necessary for regulation of rhythmic hepatic lipid metabolism. This serendipitous finding resulted from a failed loxP gene targeting approach, which deleted the DBD as an in-frame cassette, resulting in expression of a hypomorph allele, REV-ERBα-DBD^m^ (14). This striking finding revealed that only a minority of the REV-ERBα target gene spectrum was affected by loss of the DBD, and that REV-ERB recruitment to DNA elements was driven in large part by the cell lineage–determining transcription factors HNF4A and HNF6 in the liver. REV-ERBα, as a nuclear receptor, is a tractable drug target, with synthetic ligands regulating core clock function, macrophage activation, and energy metabolism (5, 17). Lack of potency coupled with low in vivo efficacy has stalled further development for REV-ERB ligands as therapeutics for human application; however, these molecules offer invaluable tools to dissect REV-ERB function.

Here we explore the hypothesis that REV-ERBα within the airway epithelial cells, and the sentinel macrophages, plays a critical role in pulmonary inflammation, and that REV-ERBα serves as a signaling relay for 2-way communication between the core circadian clock and elaboration of the inflammatory reaction. We identify distinct roles for REV-ERBα within bronchial epithelial cells and myelomonocytic cells in setting the amplitude and timing of neutrophilic inflammation in the lung. We find that REV-ERBα is dominant over REV-ERBβ, but that there is some redundancy between the 2 paralogs. We identify a pathway linking inflammatory cytokine signaling through phosphorylation to SUMOylation, ubiquitination, and degradation of REV-ERBα protein, thereby relieving inhibition of the inflammatory response. We identify and test a new REV-ERB inverse agonist, which acts to inhibit inflammatory signaling, in part by stabilizing REV-ERBα protein, rendering it resistant to SUMO and ubiquitin modification. Thus, the clock/immune interface is susceptible to pharmacological intervention.

## Results

### REV-ERBα plays a critical role in regulation of lung inflammation.

**Figure 1:**
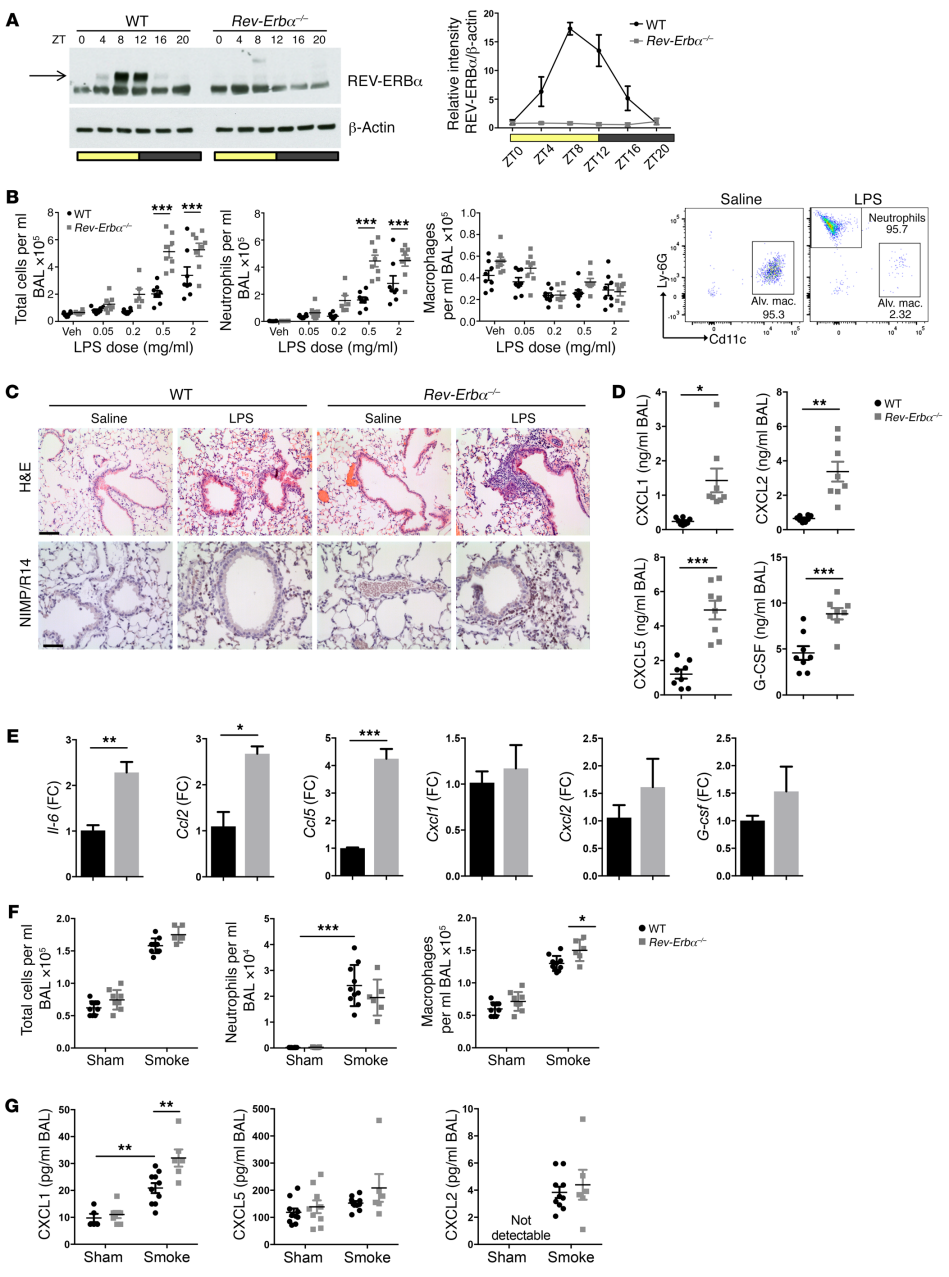
REV-ERBα plays a critical role in regulation of lung inflammation. (**A**) Whole-lung REV-ERBα protein across the day (ZT, time from lights on). REV-ERBα densitometry (mean ± SEM) was normalized to β-actin and to WT at ZT0; *n* = 5 for WT and *n* = 3 for *Rev-Erbα^–/–^* per time point. (**B**) Mice were exposed to aerosolized LPS at ZT4 and culled 5 hours later; cellular infiltrates were quantified in BAL using flow cytometry. Data presented as mean ± SEM; *n* = 6–8, ***P < 0.001 (2-way ANOVA, post hoc Bonferroni). Veh, vehicle. (**C**) H&E staining and immunohistochemistry for the neutrophil maker (NIMP/R14) of lung sections from mice after LPS challenge at 2 mg/ml. Representative of *n* = 4; scale bars: 50 μm. (**D**) Cytokine/chemokine levels in BAL fluid from mice exposed to aerosolized LPS (2 mg/ml). Representative of *n* = 8, Student’s *t* test with Welch’s correction. (**E**) Quantitative PCR (qPCR) analysis of cytokine transcripts in alveolar macrophages isolated from mice and stimulated ex vivo with LPS at 100 ng/ml for 2 hours. Data normalized to WT and presented as mean ± SEM; *n* = 3, *P < 0.05, **P < 0.01, ***P < 0.001 (Student’s *t* test). FC, fold change. (**F**) Ten-day cigarette smoke exposures were performed between ZT8 and ZT10, and animals were culled 20 hours after the last exposure. Cellular infiltrates were quantified in BAL using a hemocytometer for total cell number and cytospin for neutrophil and macrophage counts. Data presented as mean ± SEM; *n* = 6–10, *P < 0.05, ***P < 0.001 (2-way ANOVA, post hoc Bonferroni). (**G**) Chemokine levels in BAL fluid after 10-day cigarette smoke exposures. Data presented as mean ± SEM; *n* = 6–10, **P < 0.01 (2-way ANOVA, post hoc Bonferroni).

Previously, we have shown that BMAL1 deletion in the bronchial epithelium blocks circadian transcriptional cycles specifically in these cells, impacts on oxidative stress responses, suppresses both REV-ERBα and β expression, and both abolishes time-of-day variation and augments pulmonary neutrophilic inflammation (2). Conditional deletion of BMAL1 is widely used in experimental models, as this is the only core molecular element driving circadian cycles in which suppression of function leads to abolition of rhythmic transcriptional oscillations in the cell. In order to identify specific circadian-regulated proteins that directly couple to the regulation of immunity, we focused on the circadian REV-ERB transcription factors, which have been previously been identified as strong candidates for the regulation of diverse physiological functions, including rhythmic regulation of hepatic metabolism and adipose tissue (14, 18). To identify the role of REV-ERBα in circadian control of lung inflammation, we first raised a monoclonal antibody that specifically detects REV-ERBα but not its paralog REV-ERBβ (Supplemental Figure 1; supplemental material available online with this article; https://doi.org/10.1172/JCI93910DS1). This antibody revealed a marked daily pattern of REV-ERBα protein expression in whole lung, with a peak in the day around zeitgeber time ZT8–12 for WT mice, and absence of expression in knockout mice (Figure 1A). By convention, ZT0 is the time of lights-on. Protein expression was in close phase to the transcript rhythms, with peak expression between ZT8 and ZT12, suggesting a rapid transcriptional-translation process and turnover of the protein (Supplemental Figure 2A).

We tested responses of global REV-ERBα knockout (hereafter defined as *Rev-Erbα^–/–^*) and WT mice to nebulized lipopolysaccharide (LPS) at ZT4, when REV-ERBα protein is accumulating, and harvested animals 5 hours later, at peak of REV-ERBα. We observed an exaggerated neutrophilic inflammation in knockout mice (Figure 1, B and C), accompanied by significantly augmented chemokine and inflammatory cytokine responses, including CXCL5, the chemokine required to mediate the BMAL1 effect (ref. 2, Figure 1D, and Supplemental Table 1).

We have previously shown exaggerated pulmonary inflammatory responses at ZT0 compared with ZT12 (2). Therefore, we repeated the challenges at the anticipated peak and trough of this natural inflammatory response oscillation (ZT0 and ZT12). These studies again revealed an increase in neutrophilic inflammation in WT mice at ZT0 versus ZT12, but *Rev-Erbα^–/–^* mice showed augmented responses at both time points, and loss of diurnal variation (Supplemental Figure 2, B and C, and Supplemental Table 2). The most striking genotype difference was seen at ZT12, and at that time point the knockout mouse bronchoalveolar lavage (BAL) chemokines CXCL1, CXCL2, and G-CSF were elevated, but we did not see any difference in CXCL5 (Supplemental Table 2 and Supplemental Figure 2C), indicating a broader spectrum of REV-ERBα activity.

To extend these observations, we isolated alveolar macrophages from *Rev-Erbα^–/–^* mice and showed that these exhibited increased ex vivo cytokine responses to LPS, including *Il-6* (Figure 1E). However, we did not observe significant changes in *Cxcl1* and *Cxcl2* chemokines, while for *Cxcl5* the primary source in pulmonary tissues is the epithelium and not alveolar macrophages (19).

We extended these observations to other innate inflammatory stimuli and assessed responses to a single exposure of cigarette smoke, a prevalent real-world environmental challenge to the lung. No cellular inflammatory responses were detected following this transient challenge, but *Cxcl5* transcript was significantly elevated specifically in lungs of *Rev-Erbα^–/–^* mice (Supplemental Figure 2, D and E). Following multiple smoke exposures (10 days), there was mortality of 3 of the 9 *Rev-Erbα^–/–^* mice, but none of the WTs (nonsignificant by χ^2^ test). A slight increase in airway macrophages was observed in *Rev-Erbα^–/–^* mice compared with littermate controls (Figure 1F). There was an increase in CXCL1 in BAL samples from *Rev-Erbα^–/–^* mice exposed to cigarette smoke compared with littermate controls, but no differences were observed for other chemokines and cytokines in BAL samples (Figure 1G), indicating a striking specificity of response.

Because of the lack of a robust cigarette smoke phenotype, and the identification of differentially regulated eosinophil chemokines (Supplemental Table 1; IL-5, CCL11), we analyzed an allergic inflammatory challenge using ovalbumin (OVA; Supplemental Figure 3 and ref. 20). There were no significant differences in response between genotypes, suggesting that REV-ERBα had particular selectivity for innate rather than adaptive immune responses in the lung.

### Targeting Rev-Erbα in myeloid cells impairs time-of-day variation in pulmonary neutrophilic inflammation.

**Figure 2:**
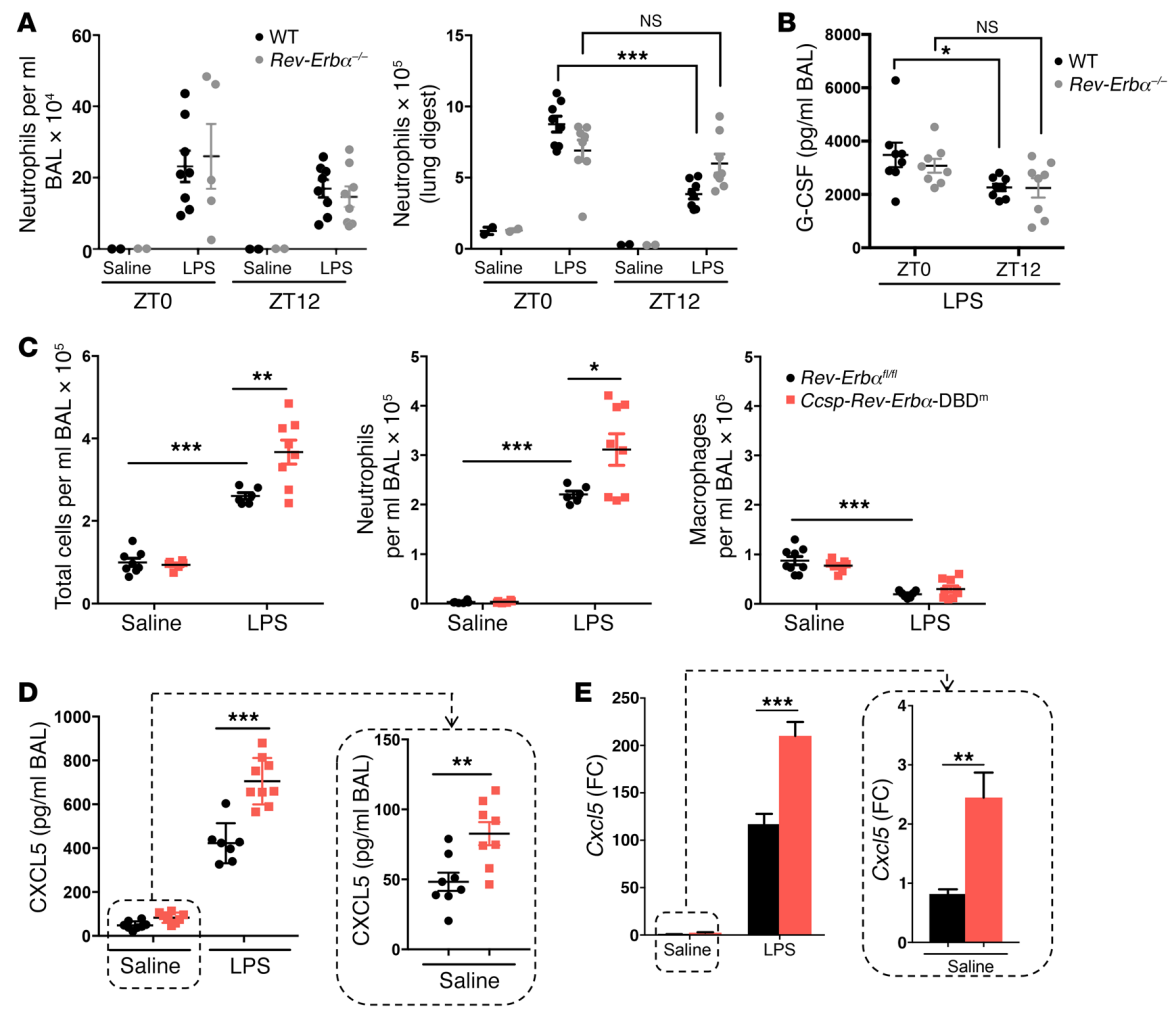
REV-ERBα in myeloid and airway epithelium regulates pulmonary inflammation. (**A** and **B**) Bone marrow cells from *Rev-Erbα^–/–^* or littermate controls were transplanted into WT recipient mice, which were then exposed to aerosolized LPS at 2 mg/ml or saline at indicated times for 20 minutes. (**A**) Neutrophil numbers in BAL samples or lung digests collected 5 hours after challenge, determined by flow cytometry analyses. (**B**) Chemokine protein levels in BAL samples. Data presented as mean ± SEM; *n* = 2 (saline) or 5–8 (LPS), *P < 0.05, ***P < 0.001 (2-way ANOVA, post hoc Bonferroni). (**C**–**E**) *Ccsp-Rev-Erbα-*DBD^m^ and littermate control mice were exposed to aerosolized LPS at 2 mg/ml or saline at ZT4 for 20 minutes. (**C**) Total cell counts in BAL samples collected 5 hours after challenge. Neutrophil and macrophage numbers in the same samples were determined by flow cytometry analyses. (**D**) Chemokine protein levels in BAL samples, measured using multiplex assay. (**E**) qPCR analysis of *Cxcl5* mRNA in lung tissues. Data normalized to saline *Rev-Erbα^fl/fl^* control group. Data presented as mean ± SEM; *n* = 5–9, *P < 0.05, **P < 0.01, ***P < 0.001 (2-way ANOVA, post hoc Bonferroni).

Both alveolar macrophages and the airway epithelium provide the first lines of defense against respiratory pathogens (21, 22). To test the role of *Rev-Erbα* in the myeloid lineage, we performed bone marrow transplant studies from global *Rev-Erbα–*null mice and from WT littermate controls (Figure 2, A and B, and Supplemental Figure 4A). Recipient mice showed only very minor changes in the circulating myeloid cell pool (Supplemental Figure 4A), but we noted that the time-of-day variation in pulmonary neutrophilia in the lung digests and in BAL G-CSF was not significant in myeloid *Rev-Erbα*–null animals (Figure 2, A and B). In this study, we directly compared the cell yields from BAL and lung digestion, as we were concerned about the variance seen in some BAL experiments (Figure 2A, left panel). Lung digestion gives more precise measurements, but excludes other measurements from the lung tissue, and for that reason we returned to BAL measures for subsequent analyses.

We next generated mice with conditional deletion of the DBD of *Rev-Erbα* in myeloid-lineage cells by crossing floxed (*Rev-Erbα^fl/fl^*) mice (14) with a *LysM^Cre/+^* strain (*LysM-Rev-Erbα-*DBD^m^) on a background of PER2::Luc in order to record circadian oscillations by bioluminescent recording. In this model, the protein retains other functions but is unable to bind directly to classical REV-ERB DNA response elements, and thereby loses its circadian clock regulating properties (14). Peritoneal macrophages derived from these mice exhibited near-total abolition of targeted *Rev-Erbα* mRNA, but expressed the truncated, DBD-deleted *Rev-Erbα* transcript (Supplemental Figure 4B), consistent with previous findings (14). We cultured circadian-synchronized peritoneal macrophages, and the targeted deletion did not impair core circadian oscillations of PER2::Luc bioluminescence (Supplemental Figure 4C).

We did not detect differences between the *LysM-Rev-Erbα-*DBD^m^ mice and littermate controls in the inflammatory response to aerosolized LPS at ZT4 (Supplemental Figure 4, D and E). To exclude the possibility that we had missed the time at which a difference was present, a phase shift in the response curve, we compared genotypes at 6-hour intervals, but again saw no difference (Supplemental Figure 4F). Further, we deleted *Rev-Erbβ* in myeloid cells, and again saw no phenotype (Supplemental Figure 4G). From this, we conclude that REV-ERBα, in a non–DBD-dependent mechanism, in myeloid-lineage cells participates in conferring time-of-day variation in neutrophilic lung inflammation, but that the increased amplitude of response observed in the global knockouts is not seen. Therefore, we concluded that another cell type is required to explain the overall impact of global REV-ERBα loss, and next examined the role of the bronchial epithelium.

### Targeted deletion of the DBD of Rev-Erbα in bronchiolar epithelial cells augments pulmonary inflammatory responses.

We targeted bronchial epithelial cells by crossing *Rev-Erbα^fl/fl^* mice with a *Ccsp^iCre/+^* line, which is selectively expressed in pulmonary bronchoepithelial-lineage cells (2). As for the mutation in myeloid cells, deletion of REV-ERBα DBD in bronchial epithelial cells did not impair circadian oscillations of PER2::Luc bioluminescence in the bronchioles (Supplemental Figure 4H). *Ccsp-Rev-Erbα-*DBD^m^ mice exhibited a marked and exaggerated neutrophilic response to LPS compared with *Rev-Erbα^fl/fl^* littermate controls (Figure 2C). To investigate the mediating pathway, we screened a panel of 23 proinflammatory cytokines/chemokines from BAL fluid by multiplex assay, and here only a single chemokine, CXCL5, was significantly increased in *Ccsp-Rev-Erbα-*DBD^m^ mice, both at basal levels and following LPS (Figure 2D and Supplemental Table 3). RNA analyses for inflammatory genes in whole lung extracts also revealed marked specificity of response for *Cxcl5* (Figure 2E). Therefore, bronchial epithelial deletion of the REV-ERBα DBD offers a similar but milder phenotype compared with the global REV-ERBα deletion. The differences seen in comparing the global loss of REV-ERBα likely reflect contributions from both the epithelium and also the myeloid lineage, and, interestingly, while the DBD of REV-ERBα is required in the epithelium for the effect on the magnitude of inflammation, it is not needed within the myeloid lineage. Aerosolized LPS challenge at ZT0 or at ZT12 further identified that the exaggerated inflammatory responses in *Ccsp-Rev-Erbα-*DBD^m^ mice were largely confined to ZT0 (Supplemental Figure 4I and Supplemental Table 4).

### Dual targeting of Rev-Erbα and Rev-Erbβ in CCSP-expressing cells abolishes diurnal rhythmicity in the airway epithelium and exaggerates inflammatory responses.

**Figure 3:**
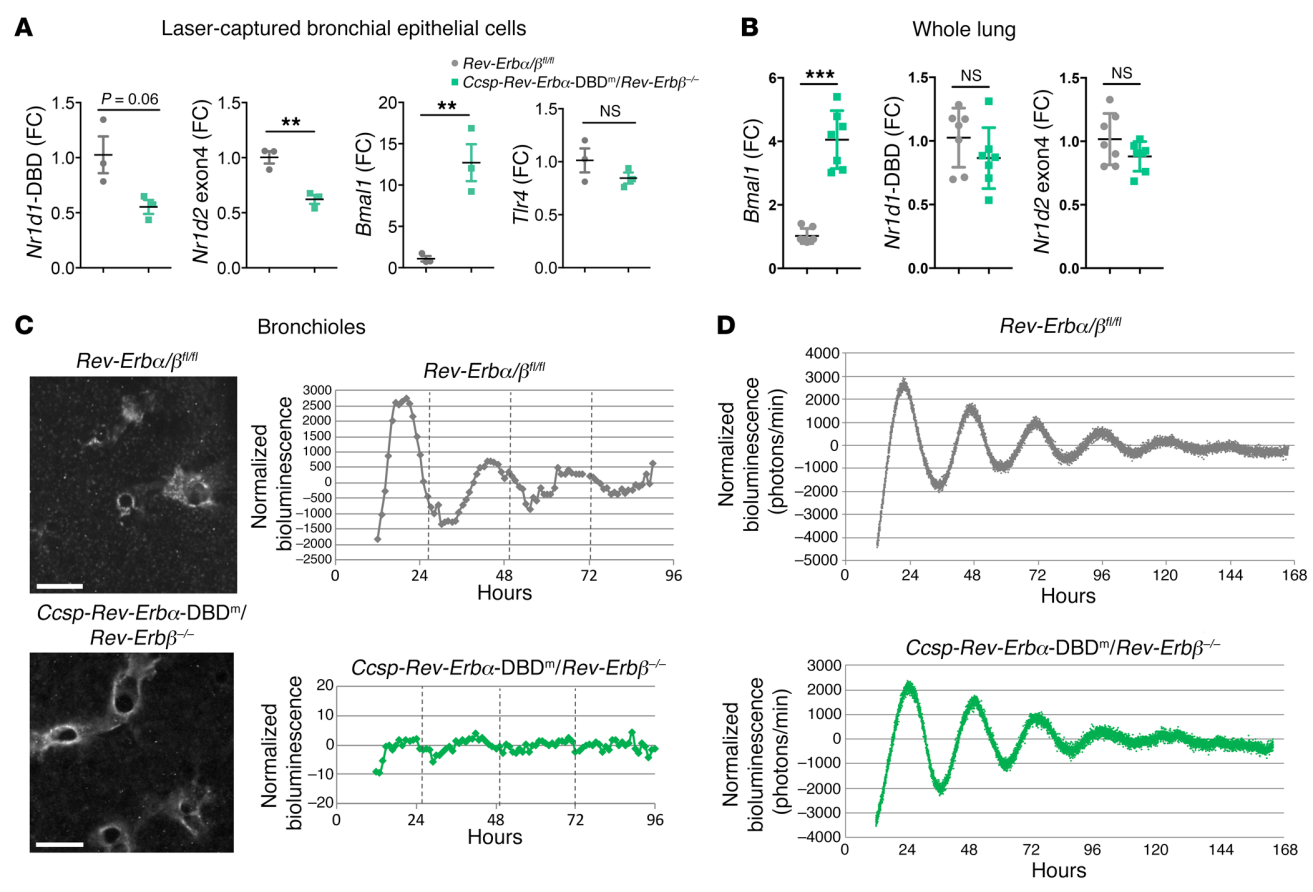
Both REV-ERB paralogs are required for circadian rhythms in the airway epithelium. (**A**) qPCR analysis of mRNA in bronchial epithelial cells, laser-captured from lung tissues collected at ZT9. Data normalized to *Rev-Erbα/β^fl/fl^* control group and presented as mean ± SD; *n* = 3, **P < 0.01, Student’s *t* test. (**B**) qPCR analysis of mRNA in whole lung collected at ZT9. Data normalized to *Rev-Erbα/β^fl/fl^* control group and presented as mean ± SEM; *n* = 7, ***P < 0.001, Student’s *t* test. (**C**) Snapshots of PER2 oscillations in bronchioles within precision-cut lung slices. Scale bars: 500 μm. Bioluminescence intensity from bronchioles was quantified, normalized to a 24-hour moving average. Traces are representative of 2 biological replicates. (**D**) Bioluminescence recordings of whole-lung PER2 oscillations in precision-cut lung slices. Photon counts per minute were normalized to a 24-hour moving average, and traces are representative of 3 biological replicates.

Both REV-ERBα and its paralog REV-ERBβ are recognized as partially redundant elements driving the core circadian oscillator, with disruption of both genes leading to loss of circadian rhythmicity (13, 23), which makes them bona fide core clock genes. To determine the effects of possible functional redundancy between the REV-ERBs, both of which require BMAL1 for expression, mice with conditional deletion of both *Rev-Erbα* DBD and *Rev-Erbβ* in bronchiolar epithelial cells (*Ccsp-Rev-Erbα-*DBD^m^/*Rev-Erbβ^–/–^*) were generated by breeding of *Rev-Erbα^fl/fl^/Rev-Erbβ^fl/fl^* mice with *Ccsp^iCre/+^* mice, with all strains bred on a PER2::Luc background. Laser capture of bronchiolar epithelial cells from lungs collected at ZT9 confirmed the efficiency of the gene targeting strategy with marked reduction in the targeted exon transcripts, accompanied by a 12-fold increase in *Bmal1* transcript in the double-mutant mice compared with the littermate controls (Figure 3A). As described in previous studies (14, 24), this led to an increase in transcript levels of a DBD-deleted *Rev-Erbα* transcript, which is translated, but no change in the expression of an exon 4–deleted *Rev-Erbβ* transcript, which is not translated, in the bronchiolar epithelial cells (Supplemental Figure 5A). As we had seen an induction of LPS response in epithelial-targeted mice, we also tested for epithelial expression of the TLR4 gene, but saw no change in expression even in the double-mutant animals (Figure 3A).

In whole lung, we did not detect transcript changes for either *Rev-Erb* gene, but there was nonetheless a marked upregulation of *Bmal1* mRNA, indicating loss of REV-ERB negative feedback (Figure 3B). Bioluminescent imaging of ectopic lung slices from *Ccsp-Rev-Erbα-*DBD^m^/*Rev-Erbβ^–/–^* mice showed disrupted circadian oscillations of *PER2::Luc* in the bronchioles (Figure 3C). This loss of rhythmicity was confined to the bronchioles, as *PER2::Luc* oscillations were retained elsewhere in the lung parenchyma (Figure 3D).

**Figure 4:**
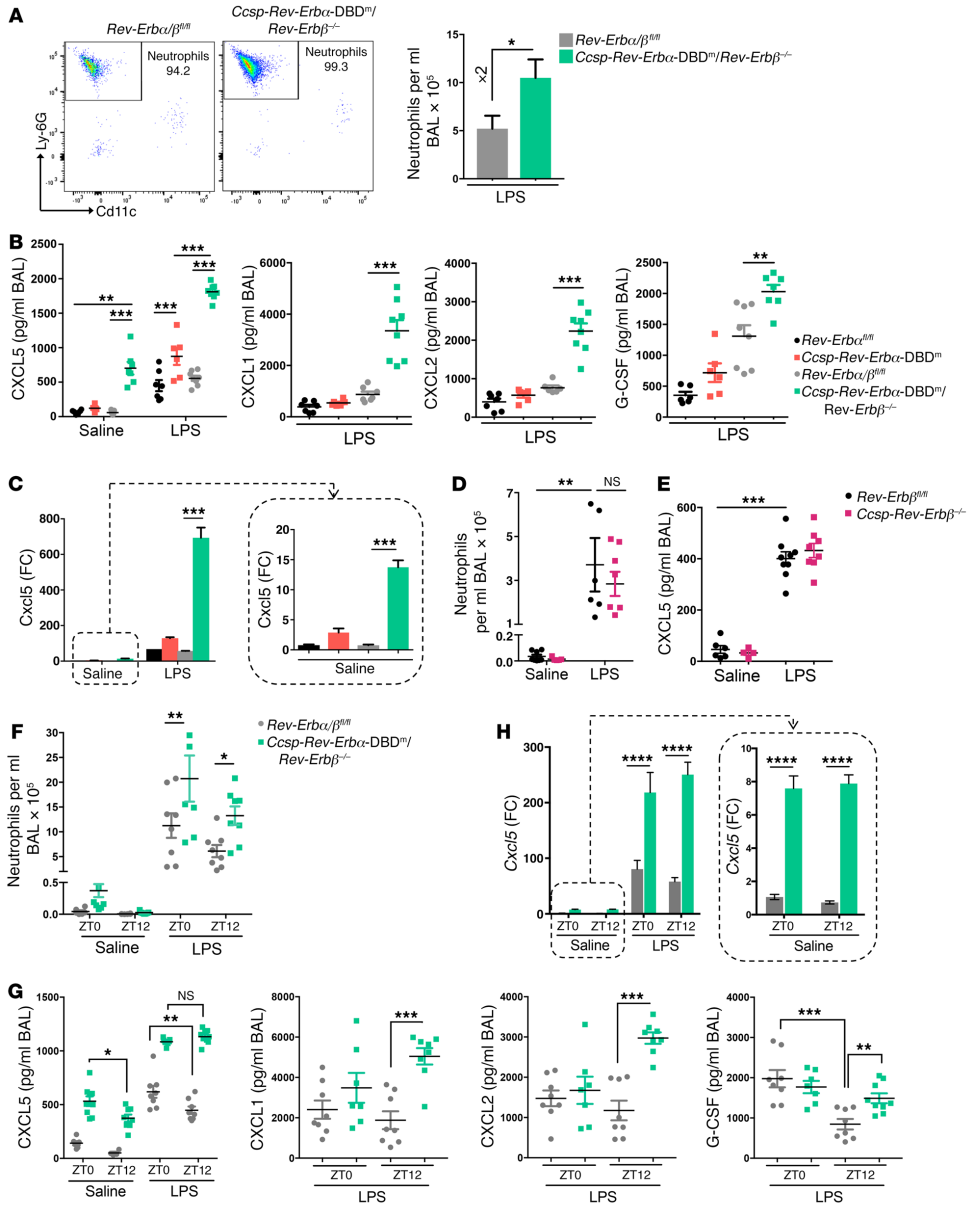
Loss of both REV-ERBα DBD and REV-ERBβ in the airway epithelium further exaggerates inflammation. (**A**) Flow analysis of neutrophils in BAL samples after aerosolized LPS (2 mg/ml) at ZT4 for 20 minutes. Data presented as mean ± SEM; *n* = 5–8, *P < 0.05 (Student’s *t* test). (**B**) Cytokine/chemokine protein levels in BAL samples after aerosolized LPS or saline at ZT4 for 20 minutes. Data presented as mean ± SEM; *n* = 5–9, **P < 0.01, ***P < 0.001 (2-way ANOVA, post hoc Bonferroni). (**C**) qPCR analysis of *Cxcl5* levels in lung tissues from the same mice as above. Data normalized to saline *Rev-Erbα^fl/fl^* control group and presented as mean ± SEM; *n* = 5–9, ***P < 0.001 (2-way ANOVA, post hoc Bonferroni). (**D**) Neutrophil numbers in BAL samples after aerosolized LPS or saline at ZT4 for 20 minutes. Data presented as mean ± SEM; *n* = 5–9, **P < 0.01 (2-way ANOVA, post hoc Bonferroni). (**E**) CXCL5 protein levels in the same BAL samples as above. Data presented as mean ± SEM; *n* = 5–9, ***P < 0.001 (2-way ANOVA, post hoc Bonferroni). (**F**) Neutrophil numbers in BAL samples collected 5 hours after aerosolized LPS challenge at ZT0 or ZT12. Data presented as mean ± SEM; *n* = 7–9, *P < 0.05, **P < 0.01 (2-way ANOVA, post hoc Bonferroni). (**G**) Cytokine/chemokine protein levels in the same BAL samples as above. Data presented as mean ± SEM; *n* = 7–9, *P < 0.05, **P < 0.01, ***P < 0.001 (2-way ANOVA, post hoc Bonferroni). (**H**) qPCR analysis of *Cxcl5* levels in lung tissues from the same mice as above. Data normalized to saline *Rev-Erbα/β^fl/fl^* control group and presented as mean ± SEM; *n* = 7–9, ****P < 0.0001 (2-way ANOVA, post hoc Bonferroni).

We next compared inflammatory responses to aerosolized LPS at ZT4 in *Ccsp-Rev-Erbα-*DBD^m^ versus *Ccsp-Rev-Erbα-*DBD^m^/*Rev-Erbβ^–/–^* mice, with littermate controls (CCSP^+/+^). Double-mutant mice exhibited larger neutrophilic responses to LPS (2-fold), compared with their littermate controls (Figure 4A), than *Ccsp-Rev-Erbα-*DBD^m^ mice (1.4-fold; Figure 2A). Only a single chemokine (CXCL5) was differentially expressed in *Ccsp-Rev-Erbα-*DBD^m^ mice in response to LPS (Figure 4, B and C, and Supplemental Table 5), consistent with the previous experiments. CXCL5 expression (both protein and transcript) was greatly increased in *Ccsp-Rev-Erbα-*DBD^m^/*Rev-Erbβ^–/–^* mice, as was the cellular inflammatory reaction to LPS. In these double-mutant mice, in addition to CXCL5, we also observed augmentation of CXCL1, CXCL2, and G-CSF production (Figure 4B) — the same 4 chemokines that showed altered expression in the global *Rev-Erbα*–null mice. Further, in *Ccsp-Rev-Erbα-*DBD^m^/*Rev-Erbβ^–/–^* mice we also observed significantly increased CXCL5 in unchallenged conditions, equivalent to that seen in control *Rev-Erbα/β^fl/fl^* littermate mice exposed to LPS (Figure 4B). In situ hybridization of lung sections from unchallenged mice demonstrated that enhanced *Cxcl5* expression in *Ccsp-Rev-Erbα-*DBD^m^/*Rev-Erbβ^–/–^* mice was confined to the bronchioles (Supplemental Figure 5B), the same structures showing *Rev-Erb* gene disruption, whereas we did not observe any change in systemic levels of CXCL5 (Supplemental Figure 5C).

The exaggerated inflammatory response observed in the double-mutant animals compared with mice with only mutation of REV-ERBα DBD could be the consequence of either an additive contribution of REV-ERBβ or dysregulation of the clock machinery in the bronchioles, the result of disruption to both *Rev-Erb* genes. To answer that question, we generated mice with conditional deletion of only REV-ERBβ in bronchiolar epithelial cells (*Ccsp-Rev-Erbβ^–/–^*) and exposed these animals and their littermate controls (*Rev-Erbβ^fl/fl^*) to an aerosolized LPS challenge at ZT4. Both groups exhibited similar inflammatory responses (Figure 4, D and E). This suggests that *Rev-Erbα* plays the dominant role in regulation of epithelial immunity in the lung, and furthermore that the more severe inflammatory phenotype observed in the double mutant is a likely consequence of a major disruption of circadian timing in these cells.

Further analysis of the double-mutant mice by time of day revealed exaggerated neutrophilic inflammation at both ZT0 and ZT12 (Figure 4F). The differences were most pronounced for CXCL5 for both the secreted chemokine and mRNA expression (Figure 4, G and H, and Supplemental Table 6), but smaller differences were also observed for CXCL1, CXCL2, and G-CSF at ZT12 in comparison with littermate controls (Figure 4G and Supplemental Table 6), indicating the impact of loss of overall clock control of inflammatory responses. These data suggest that REV-ERBα has evolved a specific role to couple rhythmic output from the circadian clock to innate epithelial immunity, while REV-ERBβ’s contribution emerges only in double knockouts, conditions that also cause loss of core cellular circadian clock oscillations, and therefore brings into play a greater diversity of clock output pathways. Importantly, we did not observe any change in the circulating neutrophil pool in response to airway impairment of both REV-ERBs (Supplemental Figure 5D). This indicates that it is local recruitment of neutrophils into the lung rather than disruption of neutrophil production and release into the circulation that explains the lung inflammatory phenotype.

### Development and biological action of a novel oxazole inverse agonist of REV-ERB.

**Figure 5:**
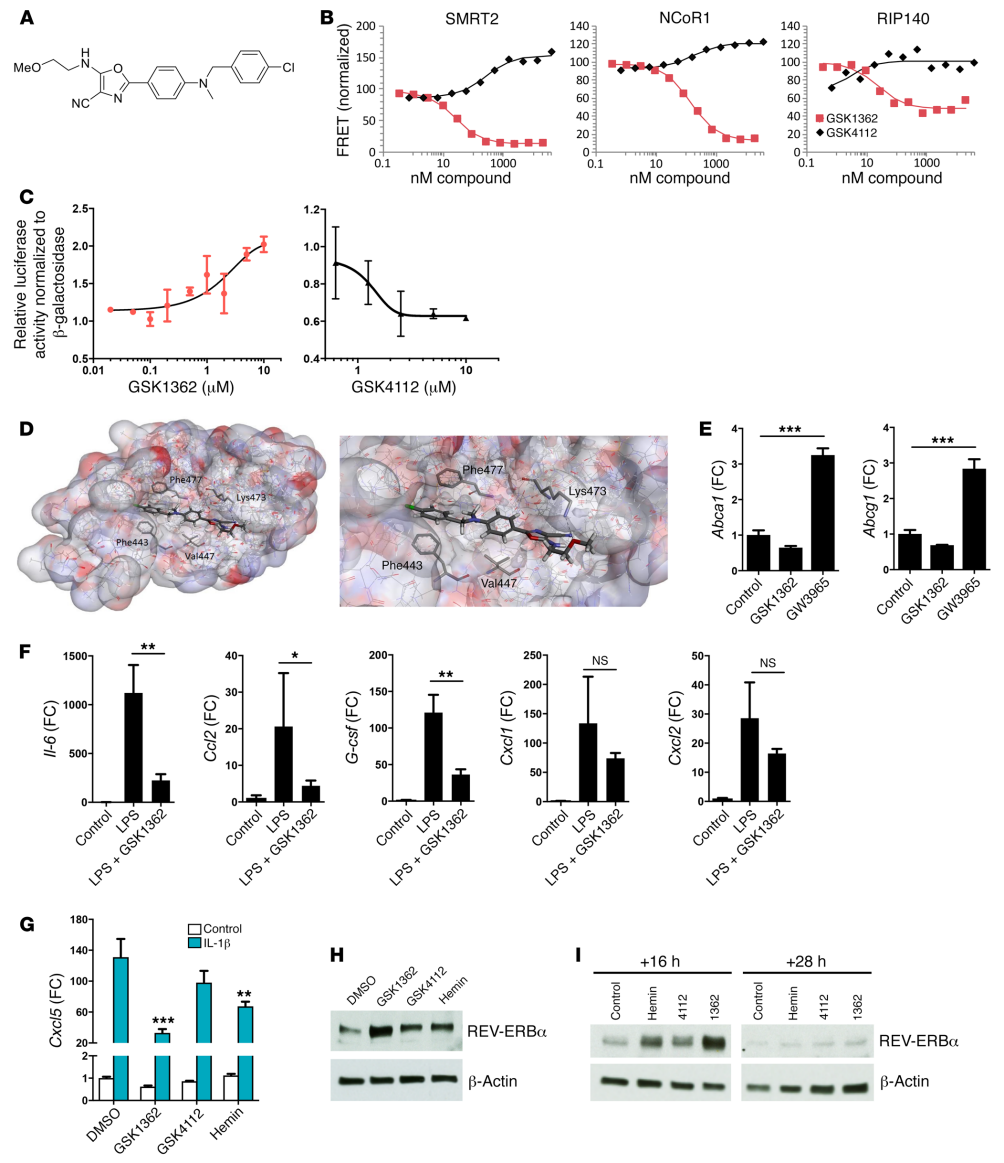
REV-ERBα ligand GSK1362 represses inflammatory genes in macrophages and epithelial cells and stabilizes REV-ERBα protein. (**A**) Chemical structure of GSK1362. (**B**) Effect of GSK1362 and GSK4112 on peptide fragment recruitment to REV-ERBα. (**C**) Cotransfection of HEK293 cells with HA–Rev-Erbα and Bmal1-Luc reporter. Cells were treated with GSK1362 or GSK4112 at different concentrations for 24 hours before luciferase assay. Values plotted relatively to 0.1% DMSO; error bars indicate mean ± SD. Data representative of *n* = 3. (**D**) Models showing GSK1362 docked in REV-ERBα ligand-binding domain. (**E**) qPCR analysis of LXR target genes in peritoneal exudate cells treated ex vivo with GSK1362 at 10 μM or GW3965, a standard LXR agonist, at 2 μM for 4 hours. Data presented as mean ± SD; *n* = 3, ***P < 0.001 (1-way ANOVA, post hoc Bonferroni). (**F**) qPCR analysis of cytokine mRNA in alveolar macrophages collected at ZT8, seeded into plates and directly treated with GSK1362 at 10 μM in the presence or absence of LPS at 100 ng/ml for 4 hours. Data presented as mean ± SD; *n* = 3, *P < 0.05, **P < 0.01 (1-way ANOVA, post hoc Bonferroni). (**G**) qPCR analysis of *Cxcl5* in LA-4 cells synchronized by serum shock and treated 16 hours later with ligands at 10 μM, followed 2 hours later by IL-1β at 1 ng/ml for 2 additional hours. Data normalized to unstimulated control cells and presented as mean ± SD; representative of *n* = 3, **P < 0.01, ***P < 0.001 (2-way ANOVA, post hoc Bonferroni). (**H**) REV-ERBα protein in LA-4 cells synchronized by serum shock and treated 16 hours later with ligands at 10 μM for 4 hours. Representative of *n* = 3. (**I**) REV-ERBα protein in NHBE cells synchronized by serum shock and treated 16 or 28 hours later with ligands at 10 μM for 4 hours. Representative of *n* = 3.

The availability of specific ligands permits new insight into nuclear receptor biology. Previous attempts to target the REV-ERBs have identified agonists with variable off-target effects on LXRs (15) and low efficacy. In pursuit of new chemical biology tools, we developed a specific ligand with inverse agonist properties: GSK3201362, hereafter referred to as GSK1362 (Figure 5A and Supplemental Figure 6A). Using an established fluorescence resonance energy transfer (FRET) assay (15), recruitment of comodulator peptide sequences to REV-ERBα protein in the presence of GSK1362 was determined, and compared with the previously reported tertiary amine agonist GSK4112 (Figure 5B). GSK1362 inhibited interaction of the REV-ERBα ligand-binding domain with peptides derived from NCoR1 and SMRT2, two repressive comodulators, characteristic actions of an inverse agonist. The interaction of a peptide derived from RIP140, a comodulator for NF-ĸB/RelA–dependent cytokine gene expression, with REV-ERBα was also repressed in a dose-dependent manner. In contrast, the agonist compound GSK4112 promoted the recruitment of NCoR1 and SMRT2 and did not regulate recruitment of the RIP140 peptide. The activity of GSK1362 was also compared with that of GSK4112 using a *Bmal1* reporter assay. GSK1362 concentration-dependently increased transcription, while GSK4112 caused inhibition (Figure 5C), suggesting an inverse agonist effect of the compound acting to relieve *BMAL1* repression by endogenous REV-ERB ligands such as heme. To gain additional information on GSK1362 engagement with REV-ERBα protein, we established a cellular thermal shift assay (CETSA), which revealed a change in REV-ERBα protein stability resulting from GSK1362 exposure (Supplemental Figure 6B). CETSA is a simple, robust, and agnostic assay, which reports changes in protein structure induced by ligand binding (25). A model for GSK1362 bound to REV-ERBα was constructed from the crystal structure of REV-ERBα bound to NCoR ID1 (26) (pdb 3N00) (Figure 5D). Key REV-ERB interactions with the ligand are driven by the highly hydrophobic ligand-binding domain of the protein and include the terminal 4‑chlorobenzyl group flanked by Phe433 and Phe477 along with the central aromatic ring interacting with Val447. The *O*-methyl ethanolamine side chain of the oxazole proved crucial for activity, which could interact through a key hydrogen bond with Lys473. Importantly, GSK1362 lacked the LXR activity that was seen in first-generation REV-ERB ligands, as it failed to induce expression of the known LXR target genes *Abca1* and *Abcg1* (Figure 5E).

We previously demonstrated that the REV-ERB agonist GSK4112 inhibited cytokine production from activated macrophages (5). We were therefore surprised to discover that REV-ERB inverse agonist GSK1362 also inhibited LPS induction of several inflammatory cytokines from alveolar macrophages (Figure 5F). In bone marrow–derived macrophages, GSK1362 inhibited *Il-6* gene expression in a REV-ERBα–dependent manner (Supplemental Figure 7A), but had no effect on *Ccl2*, *Cxcl1*, or *Cxcl2*, highlighting the complex effects of REV-ERB ligands. We also used the previously described REV-ERBα antagonist SR8278 (27), and found that this had no effect on *Il-6*. These studies do not exclude the possibility of further off-target effects of the GSK1362 ligand; therefore, we screened GSK1362 against a panel of 20 nuclear receptors, which did not reveal any significant activity (Supplemental Table 7). Therefore, we add to evidence that GSK1362 acts through REV-ERBα, but GSK1362 cannot be regarded as a chemical probe, as it may have additional targets, which remain to be defined.

Extending our analysis to bronchial epithelial cells, GSK1362 inhibited *Cxcl5* transcript induction in mouse LA-4 cells, as did, to a lesser extent, the REV-ERB natural ligand hemin (Figure 5G), providing an additional line of evidence that *Cxcl5* is a REV-ERB target gene in airway epithelial cells. In contrast, the REV-ERB ligands had no major effect on other inflammatory genes (Supplemental Figure 7B). Unfortunately, despite exhaustive efforts and trying multiple antibodies, we were not successful in determining high-confidence ChIP-Seq results for endogenous REV-ERBα in LA-4 cells, which would have helped us to uncover potential recruitment of REV-ERBα to the *Cxcl5* gene promoter or enhancer regions in airway epithelial cells.

While these studies of REV-ERB ligand action were under way, we noted a marked increase in REV-ERBα protein abundance with all ligands tested, but, more importantly, with the novel ligand GSK1362 (Figure 5H). This was also seen in human primary bronchial epithelial (NHBE) cells, which have intact circadian REV-ERBα oscillations (Figure 5I and Supplemental Figure 7, C and D). However, in NHBE cells, the 2 REV-ERB ligands GSK1362 and GSK4112 had complex and divergent effects on multiple proinflammatory cytokines upon IL-1β stimulation (Supplemental Figure 7E), again emphasizing the critical role of cell type–specific factors in determining the action of REV-ERBs, the limitations of REV-ERBs as drug targets for inflammatory diseases, and important differences in response between cell types.

### Rapid degradation of REV-ERBα protein is mediated by inflammatory stimuli, and reversed by the inverse agonist GSK1362.

**Figure 6:**
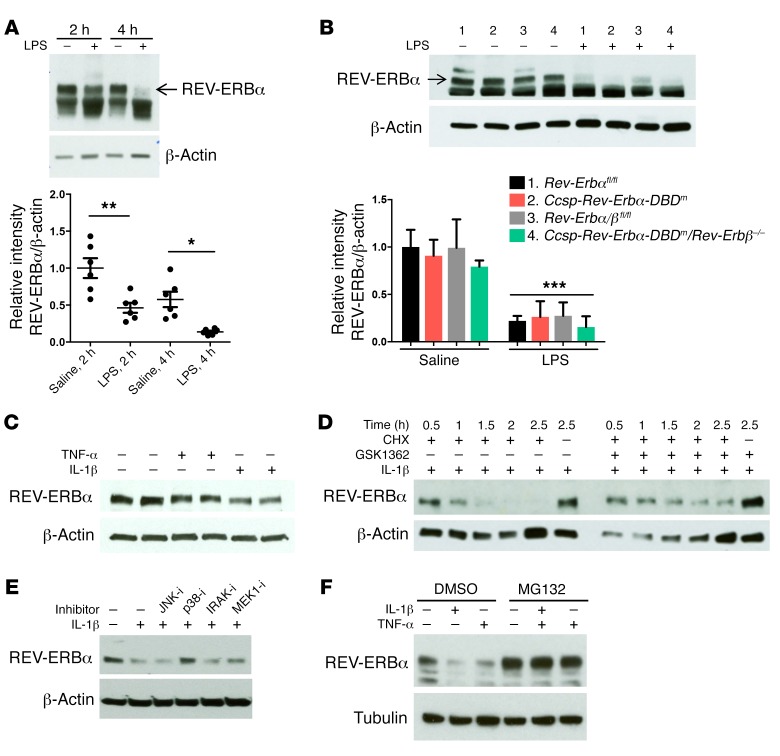
Inflammatory stimuli promote REV-ERBα degradation. (**A**) Whole-lung REV-ERBα protein in WT mice 2 or 4 hours after aerosolized LPS (2 mg/ml) or saline solution for 20 minutes at CT8 (see Methods). REV-ERBα densitometry (mean ± SEM) was normalized to β-actin and to saline at 2 hours; *n* = 6, *P < 0.05, **P < 0.01 (1-way ANOVA, post hoc Bonferroni). (**B**) Whole-lung REV-ERBα protein in mice after aerosolized LPS (2 mg/ml) or saline solution for 20 minutes at ZT4 for 5 hours. REV-ERBα densitometry (mean ± SD) was normalized to β-actin and to group 1; *n* = 3, ***P < 0.001 (2-way ANOVA, post hoc Bonferroni). (**C**) REV-ERBα protein in NHBE cells synchronized by serum shock and treated 18 hours later with TNF-α or IL-1β at 10 ng/ml for 1 hour. (**D**) REV-ERBα protein in NHBE cells synchronized by serum shock and treated 18 hours later with GSK1362 or DMSO at 10 μM followed 15 minutes later by cycloheximide (CHX) at 10 μM and IL-1β at 1 ng/ml. Cells were lysed at different times as indicated. (**E**) REV-ERBα protein in SW1353 cells synchronized by serum shock and treated 23 hours later with kinase inhibitors for 30 minutes followed by IL-1β at 5 ng/ml for 1 hour. (**F**) REV-ERBα protein in SW1353 cells synchronized by serum shock and treated 23 hours later with PBS, TNF-α, or IL-1β at 5 ng/ml for 1 hour in the absence and presence of MG132 at 5 μM. All blots are representative of at least *n* = 3.

While studies have highlighted the importance of the clock in modulation of inflammation, there is evidence that this connection is bidirectional and the inflammatory response itself can affect molecular clock pathways (28, 29). As REV-ERB ligands both repressed inflammatory responses and increased REV-ERBα protein concentration, the effect of inflammation on REV-ERBα protein was determined. Previous reports support inhibition of REV-ERBα transcription by inflammation (11, 30), but here we observed very rapid loss of REV-ERBα protein in inflamed lung tissue (Figure 6A). We also found that the REV-ERBα protein lacking its DBD also showed such degradation, again in lung tissue (Figure 6B). This was mimicked by inflammatory cytokine action in vitro (Figure 6C), and was opposed by GSK1362 (Figure 6D). Therefore, GSK1362 stabilizes REV-ERBα protein, identifying an important site of inflammatory/circadian crosstalk, an effect mediated, in part, by inflammatory activation of p38 MAP kinase (Figure 6E). REV-ERBα protein levels were greatly increased when cells were treated with the proteasome inhibitor MG132 (Figure 6F), identifying the 26S proteasome as promoting rapid REV-ERBα protein degradation.

### Posttranslational mechanisms are required for REV-ERBα degradation, and are blocked by the inverse agonist GSK1362.

**Figure 7:**
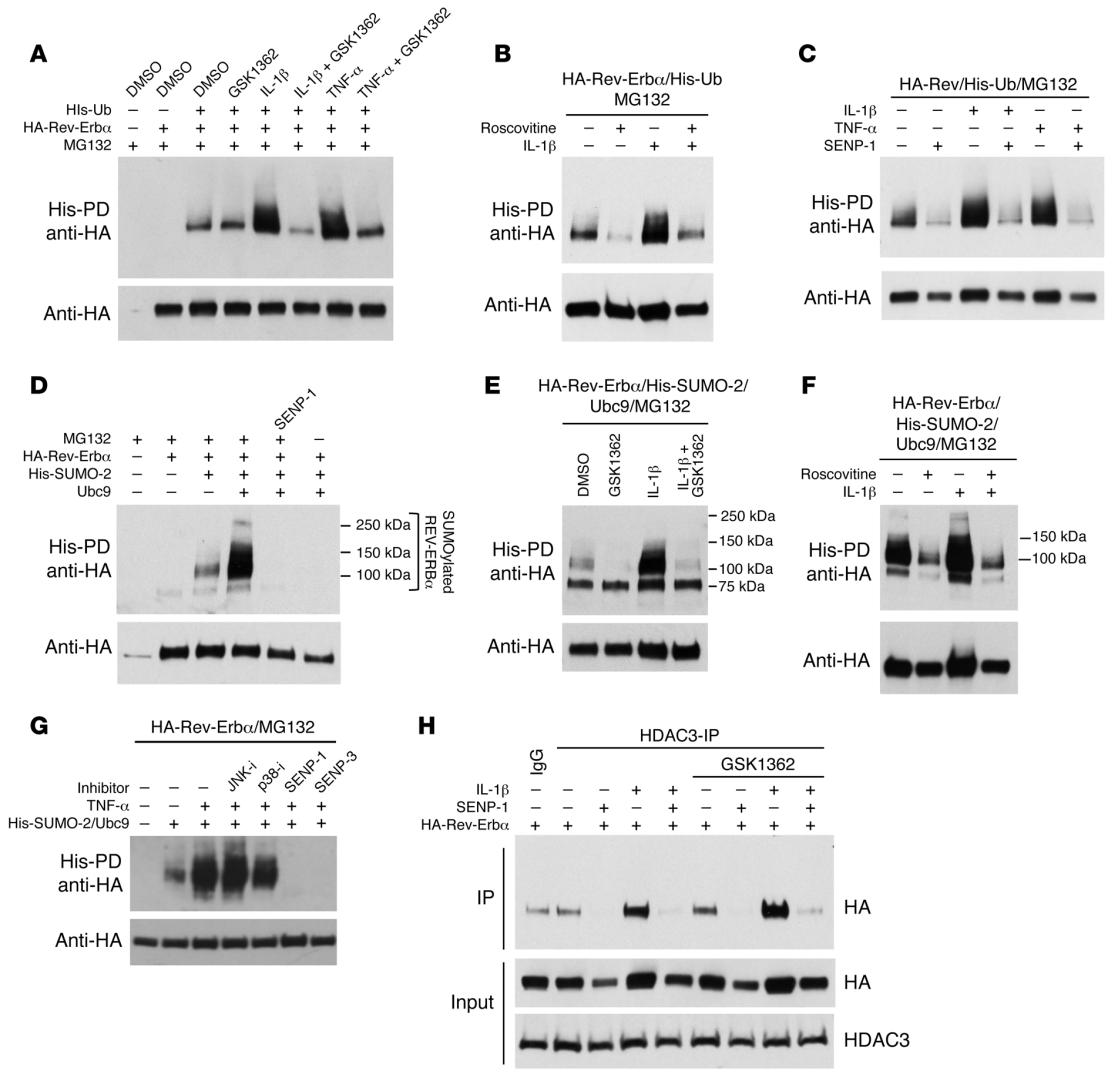
Requirement of posttranslational modifications for REV-ERBα degradation. (**A**–**C**) Ubiquitinated REV-ERBα protein in HEK293T cells transfected with HA–Rev-Erbα, His-Ub, and SENP-1 plasmids and treated with GSK1362 at 10 μM, roscovitine at 25 μM, and TNF-α or IL-1β at 5 ng/ml for 4 hours in the presence of MG132 at 5 μM. (**D**–**G**) SUMO-2 ligation to REV-ERBα protein in HEK293T cells transfected with HA–Rev-Erbα, His-SUMO2, Ubc9, and SENP-1 plasmids and treated with GSK1362 at 10 μM and kinase inhibitors and IL-1β or TNF-α at 5 ng/ml for 4 hours in the presence of MG132 at 5 μM. (**H**) Coimmunoprecipitation of HDAC3 and REV-ERBα protein in HEK293T cells transfected with HA–Rev-Erbα and SENP-1 plasmids and treated with GSK1362 at 10 μM and IL-1β at 5 ng/ml for 4 hours. All blots are representative of at least *n* = 3.

Posttranslational modifications such as phosphorylation, SUMOylation, and ubiquitination constitute an important regulatory system in nuclear receptor function. The rapid, proteasomal degradation of REV-ERBα induced by inflammatory cytokine action suggests a ubiquitination step. Indeed, REV-ERBα was rapidly ubiquitinated in response to either IL-1β or TNF-α action, an effect blocked by GSK1362 (Figure 7A). Interestingly, ubiquitination of REV-ERBα upon IL-1β treatment was also prevented by the CDK inhibitor roscovitine (Figure 7B), suggesting that the CDK1/FBXW7 pathway targeting REV-ERBα, described in previous studies (31), is activated by inflammatory cytokines. In addition to CDK inhibition impairing ubiquitination, the SUMO protease SENP-1 was also highly effective (Figure 7C), indicating that SUMOylation is a requirement for REV-ERBα ubiquitination, and prompting further investigation into REV-ERBα SUMOylation, which revealed Ubc9-dependent SUMO-2 ligation (Figure 7D). IL-1β promoted SUMO-2 ligation to REV-ERBα, whereas ligand GSK1362 blocked it (Figure 7E), and again inhibition of CDKs with roscovitine blocked SUMOylation (Figure 7F), supporting a pathway of phosphorylation followed by SUMO-dependent ubiquitination. As we had identified p38 inhibition to block inflammatory cytokine–driven REV-ERBα degradation, we looked for a p38 role in SUMOylation also, and indeed showed that p38 inhibition, but not JNK inhibition, reduced REV-ERBα SUMOylation (Figure 7G).

Inflammatory signaling also drove a SUMOylation-dependent recruitment of HDAC3 to REV-ERBα, an effect that was not disturbed by GSK1362 ligand binding (Figure 7H). The failure of ligand binding to regulate HDAC3 recruitment suggests that the principle mechanism of ligand action to suppress inflammatory cytokine expression is by stabilizing the REV-ERBα protein under conditions favoring rapid degradation. We attempted to localize the modified lysine in REV-ERBα using a mutagenesis approach, but were not able to identify a single, dominant lysine. We consider it likely that the REV-ERBα protein is multiply modified, with a complex code including phosphorylation, SUMOylation, and SUMO-dependent ubiquitination.

Analysis of REV-ERBβ modification, in contrast, revealed only a very minor increase in ubiquitination in response to IL-1β (Supplemental Figure 8A), and we could not detect SUMOylation under any circumstance (Supplemental Figure 8B). This again fits with the divergence of function, with REV-ERBα playing the dominant role in this signaling circuit.

## Discussion

The circadian clock plays a vital role in coordinating many physiological programs across tissues in anticipation of predictable changes in the environment, principally day-night. The role of such biological timing mechanisms in inflammation and immunity is being defined now, with the most prominent phenotypes centering on macrophages and innate immunity. We have previously defined the role of BMAL1, the only essential core clock component for cell-autonomous oscillations, in pulmonary inflammation and immunity. Moreover, we defined a surprising dominant role for timing circuits in the airway epithelium, rather than in the myelomonocytic lineage, by disrupting BMAL1 in these cells. However, the role of BMAL1 versus loss of timing, and the downstream pathways regulating inflammatory signaling, remained key unanswered questions. We now identify a highly specific circuit requiring the nuclear receptor and tractable drug target REV-ERBα that mediates the BMAL1 effect within bronchial epithelial cells to regulate pulmonary inflammation.

Global loss of REV-ERBα resulted in exaggerated pulmonary, innate immune responses with loss of the normal evening nadir in inflammatory response to LPS. Analysis of the myeloid cell population revealed that complete loss of REV-ERBα had a modest effect on pulmonary neutrophilic inflammation, with attenuation of the time-of-day variation. In these studies, we compared analysis of BAL with lung digest, as a means to recover immune cells from inflamed lung, and confirmed that the digest offers a more precise assay. Indeed, we only observed the loss of time-of-day variation in lung digest analysis.

Mutation of the REV-ERBα DBD in myeloid cells had no impact on pulmonary inflammatory responses in our in vivo model, which was unexpected, both because isolated alveolar macrophages lacking REV-ERBα show exaggerated LPS responses ex vivo, and because complete loss of REV-ERBα did have a phenotype. Therefore, we conclude that the REV-ERBα effect in myeloid cells is exerted through a non–DBD-dependent mechanism, akin to that documented in the liver, where DBD-independent control of lipid metabolism was seen to dissociate from the clock-regulating functions of REV-ERBα, which require binding to consensus REV-ERB *cis* elements (14). Deletion of REV-ERBβ in the myeloid lineage did not result in a lung inflammatory phenotype. In contrast, mutation of REV-ERBα DBD in bronchiolar epithelial cells resulted in increased neutrophilia and CXCL5 levels. This suggests a prominent conditioning role for the airway epithelial cells in vivo and indicates that REV-ERBα in bronchiolar epithelial cells possesses an antiinflammatory function, which is operated by its ability to bind DNA. Mice with complete loss of REV-ERBα in bronchiolar epithelial cells are not yet available but would be particularly useful to determine a potential non–DNA-binding effect of REV-ERBα in the airway epithelium upon gating of the inflammatory responses, similar to the effect observed in REV-ERBα–null myeloid cells.

As both REV-ERBα and REV-ERBβ show major physiological redundancy, the question of REV-ERBβ function required analysis. Using a double-mutant mouse model, we found a significant compensatory role for REV-ERBβ, with greatly exaggerated LPS responses, involving more chemokines, and also, intriguingly, leading to augmented CXCL5 production under basal conditions. However, we discovered that the REV-ERBα paralog is dominant over the REV-ERBβ, which could be deleted in the bronchial epithelium without a resulting inflammatory phenotype.

There has been great interest in understanding how the circadian clock affects immunity (7, 8, 32). BMAL1 deletion in the pulmonary epithelium blocks circadian control of inflammation, and augments the inflammatory response. BMAL1 plays many roles in development beyond its circadian function, and indeed postnatal BMAL1 loss results in a far milder phenotype than constitutive deletion (33). Some studies have revealed a direct role for BMAL1 acting to regulate NF-ĸB function either through enzymatic modification (9) or through regulation of microRNA (10). In our current study, we show that functional disruption/deletion of the 2 REV-ERB paralogs is proinflammatory, despite a massive increase in BMAL1 expression, with the same CXCL neutrophil chemokines and G-CSF emerging as key inflammatory mediators. These new data demonstrate a functionally important pathway from the core clock through REV-ERBα to regulate pulmonary inflammation. However, further studies will be required to establish with high confidence REV-ERBα cistrome in airway epithelial cells and uncover relevant transcriptional mechanisms.

Functional analysis of nuclear receptors can be greatly accelerated by use of regulating ligands, and previous attempts to target REV-ERB have been promising. However, early-generation ligands had significant LXR liability, prompting us to develop a new compound with no LXR action, but with a unique pattern of comodulator peptide recruitment. In vitro, GSK1362 promoted disruption of repressor peptide interaction and so is an inverse agonist, an activity supported by divergent effects on *Bmal1* promoter regulation in comparison with GSK4112, an agonist. GSK1362 was tested on both isolated macrophages and bronchial epithelial cells, with divergent and complex results, some of which may result from off-target actions. We screened GSK1362 against a 20–nuclear receptor panel, and found negligible activity. We also used a CETSA assay to gain additional information on engagement with REV-ERBα. Our sufficiency studies were necessarily limited in scope, but provided evidence that REV-ERBα was required for regulation of *Il-6*, a well-characterized REV-ERBα target gene in macrophages. Therefore, GSK1362 is a useful chemical tool, capable of binding to REV-ERBα and regulating its function. However, we do not claim GSK1362 has all the criteria for use as a chemical probe for REV-ERBα.

While investigating the mechanism of GSK1362 action we discovered a surprising ligand-dependent increase in REV-ERBα protein, suggesting that the observed chemokine repression seen with the ligand may in part be due to increased abundance of the repressor REV-ERBα protein.

We discovered that inflammation promotes degradation of REV-ERBα protein, through SUMO-dependent ubiquitination of the protein and involvement of CDKs and p38 MAP kinase. Strikingly, the inflammation-driven modification of REV-ERBα was efficiently blocked by GSK1362, which therefore resulted in an increase in REV-ERBα protein, and more efficient inflammatory mediator gene repression. Unfortunately, GSK1362 cannot be used in vivo because of adverse predicted PK characteristics. GSK1362 is therefore a useful chemical biology tool for interrogating REV-ERBα function, but whether it will be possible to make a useful human therapeutic targeting REV-ERB remains an open question. In vivo effects of other REV-ERBα ligands have been reported (34, 35), but these recent studies did not compare the effects in REV-ERBα–null mice, making it impossible to attribute the phenotypes observed to a specific action on REV-ERBα.

Taken together, our data illuminate a new circuit lying between the core circadian clock and inflammation, with both major networks converging on REV-ERBα protein, an obligate repressor of aspects of the pulmonary inflammatory response. Identification of REV-ERBα degradation as a robust early response to inflammation supports a homeostatic role in determining the inflammatory set point, limiting inflammatory activity under resting, nonstress conditions, as evidenced by the high basal production of chemokines in the bronchial epithelium of double-mutant mice (Supplemental Figure 9). REV-ERBα has been proposed as a tractable drug target for metabolic disease, and also possibly for inflammatory disease. The highly variable target gene responses seen with the new ligand suggest that any such therapeutic development would require careful consideration, not least as the pattern of response differs between cell types. However, targeting of the bronchial epithelium is tractable via an inhalation approach, although the failure of REV-ERBα disruption to impact on allergic inflammation, the major human disease burden, would temper enthusiasm. We propose that REV-ERBα degradation by inflammatory signaling offers a new pathway to explain circadian disruption in chronic inflammatory disease, and the REV-ERBα stabilization seen with ligand binding offers a new mechanism for REV-ERBα functional regulation. In addition, the emerging role of REV-ERBα as a cell type–specific mediator of timing information and control of inflammatory response gives new insights into an ancient host-defense control system.

## Methods

### Animals.

*Nr1d1^tm1Schb^* mice with global *Rev-Erbα* knockout (subsequently known as *Rev-Erbα^–/–^*) were provided by Ueli Schibler (University of Geneva, Geneva, Switzerland) (16). These animals were bred as a heterozygous colony, and offspring genotyped to identify knockout and WT animals. Conditional club cell or myeloid cell *Rev-Erbα-*DBD mutant mice (*Ccsp-Rev-Erbα-*DBD^m^ or *LysM-Rev-Erbα-*DBD^m^) were generated by breeding of *Rev-Erbα^fl/fl^* mice (*Nr1d1^tm1Ics^*; Institut Clinique de la Souris, Illkirch, France) with *Ccsp-iCre* (36) or *LysM-Cre* mice. Similarly, conditional club cell *Rev-Erbα-*DBD mutant and *Rev-Erbβ*–knockout mice (*Ccsp-Rev-Erbα-*DBD^m^/*Rev-Erbβ^–/–^*) were generated by breeding of *Rev-Erbβ^fl/fl^* mice (*Nr1d2^tm1.1Rev^*; Institut Clinique de la Souris) with *Ccsp-Rev-Erbα-*DBD^m^ mice. Tissue expression of iCre (CCSP) or Cre (LysM) was assessed by reverse transcriptase PCR as described elsewhere (2, 5). All mouse lines were subsequently crossed onto a PER2::Luc background (37). All animals were housed in a 12-hour light/12-hour dark schedule with food and water available ad libitum. In all studies, both male and female mice were used (age 6–12 weeks).

### Aerosolized LPS challenge.

In vivo LPS challenge was undertaken as described previously (2). In brief, animals were exposed to an aerosol of LPS (*E*. *coli* 0127:B8; Sigma-Aldrich) at doses ranging from 0.05 mg/ml to 2 mg/ml for 20 minutes. Animals were sacrificed 5 hours later. Bronchoalveolar lavage (BAL) was performed by instillation and removal of 1 ml aliquot of BAL fluid (10 mM EDTA in PBS with 0.1% BSA) administered via a tracheal cannula. Lung tissues were collected and snap-frozen on dry ice or were filled with paraformaldehyde and removed for histological analysis. Challenges at ZT0 and ZT4 were performed under normal light, whereas exposures at ZT12 were performed in constant darkness, using infrared goggles to view animals. Circadian time (CT) refers to time points under continuous darkness, with CT0 the start of the biological day, and CT12 the start of the biological night, and activity period in mice.

### Smoke exposure.

Cigarette smoke exposure was performed as previously described (38). Briefly, mice received a daily 2-hour nose-only exposure to 4% cigarette smoke from 3R4F cigarettes (College of Agriculture, Reference Cigarette Program, University of Kentucky, Lexington, Kentucky, USA). During exposure to smoke or air only (sham controls), mice were maintained in restraining tubes containing stainless steel nose cone inserts. BAL samples and lung tissues were collected as previously described.

### Bone marrow transplant study.

Recipient mice (B6 Cd45.1, Pep Boy; The Jackson Laboratory) were placed on Baytril antibiotic (Sigma-Aldrich) for 2 weeks before and 4 weeks after radiation exposure. Donor mice (*Rev-Erbα^–/–^* or littermate controls) were culled by cervical dislocation, and femurs/tibiae were removed. Bone marrow was flushed out using Iscove’s Modified Dulbecco’s Media (supplemented with 2% FCS) and suspended in red blood cell lysis buffer (Roche). The remaining cells were depleted of T cells using a CD90.1 Positive Selection Kit (EasySep). Remaining donor cells were counted and resuspended to 2 × 10^7^ such that each mouse received a tail vein injection of 4 × 10^6^ cells in 200 μl. Recipient mice were then given 2 doses of radiation of 5.5 Gy separated by 2 hours. Recipient mice then had 200 μl of donor cells injected into the tail vein. Mice were weighed daily after injections and left for 3 months to allow for full immune reconstitution prior to aerosolized LPS exposure.

### BAL cytokine analysis, cell counts, and flow cytometry.

BAL samples were centrifuged and the supernatant utilized for cytokine and chemokine analysis using the Magnetic Luminex Assay (R&D Systems). The cell pellets were resuspended in 200 μl PBA (PBS with 1% BSA and 0.1% sodium azide) for subsequent total cell count and analysis by flow cytometry. In brief, 19 μl resuspended cells were mixed with 1 μl of fluorescent dye solution 18 (ChemoMetec), and total cell number was assessed using NucleoCounter NC-250 (ChemoMetec). Frequency of neutrophils and macrophages was assessed by flow cytometry using anti–Ly-6G (Gr-1)–Alexa Fluor 488 and anti-CD11c–APC antibodies to detect neutrophils and alveolar macrophages, respectively (2). For flow cytometry analyses on lung digests, the left lobe of the lung was placed in 1 ml RPMI (Sigma-Aldrich) containing Liberase (Sigma-Aldrich) and DNase I (Promega) and chopped up with scissors. The homogenate was placed at 37°C for 30 minutes on an orbital shaker. Digestion was stopped with the addition of 1 ml RPMI containing 10 mM EDTA (Sigma-Aldrich). Homogenate was passed through a 70-μm sieve (Corning) and cells pelleted at 300 *g* for 5 minutes at 4°C. Pellets were resuspended in red blood cell lysis buffer. Remaining cells were pelleted, counted, and plated at 1 × 10^6^ cells for flow cytometry.

### Bronchiolar epithelial cell laser capture.

The trachea was cannulated postmortem and lungs inflated with 1 ml PBS/OCT mixture (1:1). The trachea was tied off, and the lungs and heart were removed en bloc and snap-frozen. Ten-micrometer sections were cut on the cryostat, and 3–4 sections were placed per polyethylene naphthalate (PEN) membrane slide (3 slides per animal). Before laser dissection, specimen slides were taken out of dry ice and placed into alcoholic solutions (100% ethanol for 1 minute, 75% ethanol 3 dips, 50% ethanol 3 dips, 95% ethanol 30 seconds, 100% ethanol 30 seconds, 100% ethanol 2 minutes). A Leica LMD 6500 laser-capture microdissection machine was used to cut regions around the bronchiolar airways. For tissue collection, 0.5-ml thin-wall PCR tubes were used in the tissue collector, with 30 μl lysis buffer (TRK buffer supplemented with β-mercaptoethanol, MicroElute Total RNA kit; Omega Bio-tek). Samples were then stored at –80°C prior to RNA extraction using MicroElute Total RNA kit, following the manufacturer’s instructions.

### Lung histology.

Paraformaldehyde-fixed lung tissue was processed and embedded, and 5-μm sections were mounted onto slides. Sections were used for routine H&E staining. Immunohistochemical staining was carried out using antibody raised against NIMP/R14 (to detect neutrophils; Abcam Ab2557); sections were counterstained with hematoxylin. Slides were viewed using a Leica DM2000 microscope and images captured using a Leica IC90E digital camera and software.

### Ectopic lung slices.

Precision-cut ectopic lung slices (275 μm) were prepared as previously described (39). After washes to remove residual agarose, slices were placed onto cell culture inserts (Millicell) within 35-mm dishes containing 1 ml recording medium and sealed with a coverslip. Dishes were then transferred to a 37°C incubator housing the photomultiplier tubes (PMTs; H6240 MOD1; Hamamatsu Photonics) or placed under a self-contained Olympus LV200 luminescence microscopy system fitted with a cooled Hamamatsu C9100-13 EM-CCD camera (Olympus) as previously described (40). Individual regions of interest were delineated using ImageJ software (version 1.41o; NIH).

### Peptide recruitment profiles.

These experiments were undertaken as previously described (15).

### Bmal1 luciferase assay.

HEK293 cells were transfected with HA-tagged Rev-Erbα, a luciferase reporter driven by the *Bmal1* promoter, and a β-galactosidase reporter using polyethylenimine (PEI) (3:1 vol/wt ratio) (41) and left overnight. Cells were treated with GSK1362 at different concentrations or with 0.1% DMSO for 24 hours followed by the luciferase assay using the Dual-Light Luciferase and β-Galactosidase Reporter Gene Assay System (Thermo Fisher Scientific) according to the manufacturer’s instructions. Luciferase activity was normalized to β-galactosidase reading for each sample.

### His purification.

HEK293 cells were transfected with 0.5 μg HA-tagged Rev-Erbα, 0.75 μg His-ubiquitin, 0.75 μg His–SUMO-2, 0.5 μg SENP-1, and 0.25 μg Ubc9 using PEI (3:1 vol/wt ratio) (41) and left overnight. Cells were treated with 5 μM MG132 (Sigma-Aldrich), 10 μM GSK1362 ligand, IL1-β, or TNF-α as indicated. Cells were lysed in either RIPA buffer (50 mM Tris-Cl pH 7.4, 1% NP-40, 0.25% sodium deoxycholate, 150 mM NaCl, 1 mM EDTA) supplemented with protease and phosphatase inhibitors for input samples or guanidinium/HCl buffer I (6 M guanidinium/HCl, 0.1 M Na_2_HPO_4_/NaH_2_PO_4_, 0.01 M Tris, pH8) for His-purified samples. Lysates for His purification were first sonicated using EpiShear Probe Sonicator and cell debris cleared by centrifugation. Supernatants were added to Ni-NTA Agarose beads (Qiagen), previously washed with buffer I and partially bound with BSA at 50 ng/μl (Sigma-Aldrich). Imidazole (5 mM) and β-mercaptoethanol (10 mM) were also added, and samples were incubated by rotation at room temperature for 2–3 hours. Beads were washed twice with buffer I supplemented with 5 mM imidazole (rotation at room temperature for 20 minutes followed by centrifugation at 400 *g* for 2 minutes to remove supernatant). Beads were then washed with urea buffer II (8 M urea, 0.1 M Na_2_HPO_4_/NaH_2_PO_4_, 0.01 M Tris, pH 6.3) containing 5 mM imidazole (rotating for 10 minutes), followed by another wash with buffer II containing 5 mM imidazole and 0.2% Triton X-100 (Sigma-Aldrich). Beads were finally washed with PBS, and after centrifugation at 100 *g*, supernatants were discarded. Beads were mixed with SDS loading dye containing 10% β-mercaptoethanol and boiled 10 minutes at 70°C. Samples were stored at –80°C until run for Western blotting.

### Immunoprecipitation.

HEK293 cells were transfected with 1 μg HA-tagged REV-ERBα and/or 1 μg SENP-1 using PEI (3:1 vol/wt ratio) and left overnight. Cells were treated with 10 μM GSK1362 ligand and/or 5 ng/ml IL-1β for 4 hours. Cells were lysed in either RIPA buffer supplemented with protease and phosphatase inhibitors for input samples or IP lysis buffer (150 mM NaCl, 20 mM Tris-HCl, 10% glycerol, 1% Triton X-100, 1 mM PMSF, 10 mM *N*-ethylmaleimide (NEM), PhosSTOP (MilliporeSigma), complete EDTA-free protease inhibitor cocktail) on ice and cell debris cleared by centrifugation. One microgram of anti-HDAC3 antibody (Santa Cruz Biotechnology H-99) or 1 μg of rabbit IgG was incubated with protein lysates for 1 hour on a rotating wheel at 4°C. Antibody complexes were captured by addition of beads (MagReSyn Protein A; ReSyn Biosciences) for 45 minutes at 4°C. Beads were washed 3 times with IP lysis buffer, and then boiled for 10 minutes in SDS loading dye containing 10% β-mercaptoethanol. Beads were cleared using magnetic separator, and supernatants were ready for electrophoresis.

### Generation of REV-ERBα monoclonal antibody.

The GSK6F05-2 antibody (Biocat 137359) was generated by conventional 3-month immunization of SJL mice, with His-tagged Rev-ErbA α ligand-binding domain (aa 281–614), purified from *E*. *coli*. Protein-specific monoclonal antibodies were identified by ELISA and Western blot screening of single-cell-derived clones.

### CETSA assay.

HEK293 cells were transfected with HaloTag-Rev-Erbα (as described above, 10 μg plasmid per 10-cm dish). Cells were treated with GSK1362 or DMSO (0.1% vol/vol) for 1 hour before the media was replaced with 1 ml PBS (containing protease inhibitor cocktail). One-hundred-microliter aliquots of cells (prepared without further washing in PBS) were heated (from 40°C to 58°C using a thermocycler) for 3 minutes and then incubated at room temperature for a further 3 minutes. Following 2 freeze-thaw cycles (using dry ice, and a thermocycler at 25°C), 2 microliters of 10% (vol/vol) Triton X-100 was added and the lysate vortexed. Cell lysates were centrifuged at 20,000 *g* for 20 minutes (4°C) to pellet insoluble cell debris. Supernatants were incubated with Halo-ligand (Alexa Fluor 660; Promega) for 15 minutes at room temperature before 4× lithium dodecyl sulfate (LDS) loading buffer and 100 mM DTT were added. After electrophoresis, gels were visualized using an Odyssey CLx infrared imaging system. Western blotting was carried out with PVDF (Immobilon-FL Membrane; Merck Millipore) and an anti–lamin B1 (1:1,000; Proteintech) antibody. Blots were visualized with an anti–rabbit CF800 secondary antibody (1:20,000; Biotium) and an Odyssey CLx imager.

### Statistics.

Values are expressed as mean ± SEM or mean ± SD as stated. Data were analyzed using GraphPad Prism 6.0. Parametric statistical analyses were applied when data showed normal distribution; otherwise nonparametric tests were used. The statistical analysis was conducted at 95% confidence level, with a *P* value less than 0.05 being considered statistically significant (***P ≤ 0.001, **P ≤ 0.01, *P ≤ 0.05). PMT recordings were normalized to a rolling average before plotting as a function of time; period analysis was carried out using RAP software 50, and Cosinor analysis was carried out using Cosinor.exe version 2.3 (http://www.circadian.org/softwar.html). All animals were randomized to treatment and control groups, and investigators were blinded to genotype and treatment.

### Study approval.

All animal studies were ethically reviewed and carried out in accordance with standards defined by the Animals (Scientific Procedures) Act, United Kingdom, 1986 or European Directive 86/609/EEC and the GlaxoSmithKline Policy on the Care, Welfare and Treatment of Animals.

Additional methods are described in Supplemental Methods online.

## Author contributions

MP, YS, ASIL, and DWR conceived the project. MP, JEG, ASIL, and DWR designed the experiments, and MP, ASIL, and DWR wrote the paper. JEG performed some in vivo and ex vivo experiments in REV-ERBα–knockout mice, and MP performed the majority of the experiments and downstream analyses. TWH and S. Brown performed the bone marrow transplant study, and TWH participated in some of the in vivo experiments. NB managed the transgenic animal colonies and provided technical help with in vivo experiments. RV performed the isolation of the bronchial cells by laser capture, lung section bioluminescence recordings, and technical help with in vivo experiments. TP and BG contributed to the work on posttranslational modification and degradation of REV-ERBα. BS completed studies of in situ hybridization. DHJ, JWP, JPT, S. Bresciani, NCOT, AWJC, RPT, DG, WZ, and TMW performed all the chemistry around GSK1362 and its characterization. DAD led on the development of the antibody GSK6F05. ASM supervised the bone marrow transplant study. BB and PLP undertook the cigarette smoke studies.

## Supplementary Material

Supplemental data

## Version 1 03/13/2018

In-Press Preview

## Version 2 04/30/2018

Electronic publication

## Version 3 06/01/2018

Print issue publication
