# Supplementary material for: Circadian clock component REV-ERBα controls homeostatic regulation of pulmonary inflammation
Source: J Clin Invest. 2018 Apr 30;128(6):2281–96. doi: 10.1172/JCI93910 (PMC5983347; doi:10.1172/JCI93910)
Supplement: Supplemental data [file jci-128-93910-s172.pdf]

Supplemental information

Supplemental Experimental Procedures

Ovalbumin challenge

Mice were sensitized with OVA (10μg administered with aluminium hydroxide) by i.p injection (0.2ml) on days 1 and 14. From day 24, mice were administered OVA (50μg in 50μL; n= 10-12) or saline (control; n=7-8) via an intranasal route for three consecutive days. On day 27, airway hyper-reactivity to increasing doses of inhaled serotonin (5HT; 1mg/ml, 3mg/ml and 10mg/ml) was determined in each mouse, using established non-invasive methods. Mice were sacrificed the following day, BAL collected and lung tissue fixed as described in the main experimental procedures.

In situ hybridization

Lung tissue was collected from *Ccsp-Rev-Erbα*-DBD^{m}/*Rev-Erbβ*$^{-/-}$ mice and littermate controls at ZT9 and ZT21. The trachea was cannulated post-mortem and lungs inflated with 1 ml PBS/OCT mixture (1:1). The trachea was tied off, and the lungs and heart were removed *en bloc* and snap frozen. Preparation of tissue sections, radiolabelled probes and hybridisation was performed as previously described (Gibbs et al., 2014).

Neutrophil counts in blood samples

Blood was collected and mixed 1:1 with 3.9% sodium citrate to prevent from coagulation. 5 μl of blood/sodium citrate was mixed with 45 μl solution 17 (ChemoMetec, Denmark) and incubated at 37°C for 10 minutes before measurement of total cell numbers using NucleoCounter® NC-250™ (ChemoMetec, Denmark). Frequency of neutrophils was assessed by flow cytometry. In brief, cells were incubated with anti-CD45-Pacific Blue, anti-CD11b-PerCP/Cy5.5, anti-Ly-6G (Gr-1)-AlexaFluor-488 and anti-CD11c-APC antibodies. After red blood cells lysis, cells were fixed in 3.6% formaldehyde solution. Cell suspensions were run on BD Biosciences LSR II flow cytometer along with FACSDiva software to collect data and FlowJo software to analyse cell populations. Neutrophil population was selected as CD45^{+}/Ly-6G^{+}/CD11b^{+} cells.

Primary cell culture

Alveolar macrophages were isolated from the bronchoalveolar space by lavage with BAL fluid (2 × 1 ml). The retrieved fluid was pooled from 3-4 mice, centrifuged and the pellet resuspended in RPMI containing Penicillin/Streptomycin (P0781, Sigma – 1% v/v) and plated out for cell culture. Flow cytometry analyses on these attached cells revealed that they were 97 to 99% alveolar macrophages. Cells were directly treated with ligand GSK1362 at 10μM and/or LPS at 100ng/ml for indicated times. Peritoneal exudate cells (PECs) were collected by injecting and retrieving twice 5 ml of ice cold PBS (with 3% FBS) into the peritoneal cavity. Cells were centrifuged, resuspended in RPMI containing Penicillin/Streptomycin and plated out. After 2 h, nonadherent cells were removed by gentle washing and the following day cells were treated as for alveolar macrophages. Bone marrow derived macrophages (BMDM) were isolated from mouse legs. The hind legs were detached, the muscle and skin removed and the bone cleaned before being placed into ice cold PBS. This procedure was repeated with the fore legs. The bone marrow was disrupted by pipetting up and down, centrifuged at 1,600 rpm for 6 minutes and the supernatant discarded. Cells were re-suspended in culture medium consisting of DMEM supplemented with 10% FBS, 1% Penicillin/Streptomycin and macrophage colony stimulation factor (M-CSF) (Affymetrix eBioscience) at a concentration of 50ng/ml. Cells were then plated outand culture medium was changed every 3 days for at least a week in order to let the cells differentiate into macrophages.

Primary normal human bronchial epithelial cells

Primary NHBE cells were purchased from Lonza (Walkersville, MD) and cultured as monolayers in serum-free bronchial epithelium growth medium supplemented with growth factors (BEGM, Lonza). Subculture reagents (Reagentpack, Lonza) were used according to the supplier's instructions. NHBE cells were seeded in 24-well culture plates at 5-7×10^{4} cells/well and allowed to attach overnight before synchronization with 50% FBS in BEGM (1 h). Cells were then treated as indicated and lysed for RNA or protein analyses. Cells from the same batch of culture were pooled for each individual experiment and never used after passage 5. The cells from the same donor were used in all assays for assay consistency.

Cell line culture

LA-4 cells are epithelial-like cells isolated from an urethan-induced lung adenoma of a 28 week old A/He strain mouse. LA-4 cells, purchased from Sigma, were expanded, cryopreserved, and cultured as monolayers in standard tissue culture flasks (Costar) in Ham's F12 medium supplemented with 2mM Glutamine, 1% Non-Essential Amino Acids (NEAA) and 15% FBS. For experiments, LA-4 cells were seeded at 1×10^{5} cells/well in 24-well culture plates and allowed to attach overnight before synchronization with 50% FBS in serum-free

culture medium (1 h) and treatment. HEK 293 cells, purchased from European Collection of Authenticated Cell Cultures (ECACC), were expanded, cryopreserved, and cultured as monolayers in standard tissue culture flasks (Costar) in Dulbecco's Modified Eagle's Medium (DMEM) supplemented with 10% FBS. For experiments, HEK 293 cells were seeded at 4×10^5 cells/well in 12-well culture plates or at 8×10^5 cells/well in 6-well culture plates and allowed to attach overnight before any treatment.

Q-PCR analysis

RNA was extracted from tissue using Trizol Reagent (tissue) or the RNeasy method (Cells; Qiagen). After DNase treatment, cDNA was prepared (RNA to cDNA kit, Life technologies). Q-PCR was performed using commercial Taqman primer/probe mixes (Life technologies) or bespoke primers and probes (sequences available on request). Amplification reactions were performed in duplex using eukaryotic 18s rRNA (VIC/MGB Probe, Life technologies) as endogenous control, and the primer/probe set of the gene of interest. The relative changes of gene expression were calculated using the following formula: Fold change in gene expression, $2^{-\Delta\Delta Ct} = 2^{-[\Delta Ct\ (treated\ samples)\ -\Delta Ct\ (untreated\ control)]}$, where ΔCt = Ct (detected gene) - Ct (18s rRNA) and Ct represents threshold cycle number.

Western blotting

Lung tissue and cells were lysed in RIPA buffer supplemented with protease and phosphatase inhibitors. Protein lysates from tissues were prepared using a tissue Ruptor (Qiagen) and 2 µl of benzonase nuclease (Novagen) were added. Cell debris were cleared by centrifugation. Lysates were boiled for 10 minutes in SDS loading dye containing 10% β-mercaptoethanol (v/v) (Sigma) and electrophoresed on Mini Protean TGX Precast Gels 4-15% (BioRad). Resolved proteins were transferred to a 0.2 µm pore size Protran nitrocellulose membrane for 70 minutes then rinsed with Tris-HCl pH 7.6 buffered saline (TBS) solution supplemented with 0.1% Tween-20 (TBST). Membranes were blocked (5% skim milk powder in TBST) for 1 hour at room temperature and then incubated with mouse monoclonal GSK6F05 anti-REV-ERBα, anti-HA (Roche, 12CA5) or anti-β-ACTIN (Abcam, ab8227) antibodies overnight at 4°C. Membranes were washed 3 × 10 minutes with TBST and secondary HRP-linked antibodies (GE Healthcare) were incubated for 1 hour. After 3 × 10 minute TBST washes immunoreactive bands were detected using Supersignal West Dura (ThermoScientific) and chemiluminescence visualised on Kodak BioMax MR or XAR Film.

Kinase inhibitors

JNK and p38 inhibitors were purchased from Sigma (SP600125, SP239063) and used at 10µM and 20µM respectively. IRAK1/4 and MEK1 (U0126) inhibitors were purchased from Calbiochem and used at 20µM and 5µM respectively.

Synthesis of GSK1362

Commercially available solvents and reagents were used without further purification. Flash chromatography was carried out using Merck Kieselgel 60 H silica or Matrex silica 60 unless stated otherwise. Analytical thin layer chromatography was carried out using aluminium-backed plates coated with Merck Kieselgel 60 GF254 that were visualised under UV light (at 254 and/or 360 nm). Nuclear magnetic resonance (NMR) spectra were recorded in $CDCl_3$ at 18 °C unless stated otherwise and were reported in ppm; J values were recorded in Hz and multiplicities were expressed by the usual conventions. Automated column chromatography was performed on pre-packed silica gel columns (30-90 mesh, IST) using a Biotage SP4 or on a reverse-phase Sunfire C18 column. LCMS analysis was carried out on H2Os Acquity UPLC instrument equipped with a BEH column (50 mm x 2.1 mm, 1.7 µm packing diameter) and H2Os micromass ZQ MS using alternate-scan positive and negative electrospray. Analytes were detected as a summed UV wavelength of 210–350 nm. In vacuo refers to evaporation at reduced pressure using a rotary evaporator and diaphragm pump, followed by the removal of trace volatiles using a vacuum (oil) pump.

2-Amino-3,3-dichloroacrylonitrile 3

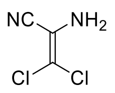

To a solution of 2,2-dichloroacetonitrile (4.95 mL, 61.5 mmol) and 2-hydroxy-2-methylpropanenitrile (6.20 mL, 67.7 mmol) in 10:1 MeCN/Et_2O (82.0 mL) was added potassium cyanide (0.08 g, 1.2 mmol) at 0 °C. Following completion of addition, the reaction mixture was left to stir overnight (16 h) at room temperature. The volatiles were removed under reduced pressure, and the residue purified by automated column chromatography (0–50% EtOAc–cyclohexane over 40 min) to give **3** as an off-white solid, (4.55 g, 54%); ^{1}H NMR (400 MHz, DMSO

d6) δ 5.99 (2H, br s); ^{13}C NMR (101 MHz, DMSO d6) δ 118.1, 113.8, 103.9; mass not seen by LCMS due to decomposition.

4-Bromo-*N*-(2,2-dichloro-1-cyanovinyl)benzamide 4

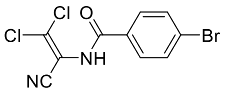

A solution of **3** (3.00 g, 21.9 mmol) in dry CH_2Cl_2 (6.0 mL) was cooled to 0 °C under a nitrogen atmosphere. 4-Bromobenzoyl chloride (5.05 g, 23.0 mmol) and aluminium chloride (3.07 g, 23.0 mmol) were added sequentially and the mixture stirred for 15 min at 0 °C, followed by 2 h at room temperature. The volatiles were removed under reduced pressure and the resultant residue dissolved in EtOAc (200 mL), washed with water (2 × 200 mL), brine (75 mL) and concentrated under reduced pressure to a volume of around 50 mL. Cyclohexane (150 mL) was added slowly, and the resulting precipitate collected and washed with cold EtOAc (10 mL) to give **4** as a colourless solid (4.12 g, 59%); ^{1}H NMR (400 MHz, DMSO d6) δ 10.74 (1H, s), 7.87 (2H, d, J = 7.0 Hz), 7.79 (2H, d, J = 7.0 Hz); ^{13}C NMR (101 MHz, DMSO d6) δ 164.5, 134.8, 131.8, 130.8, 130.0, 126.8, 113.0, 110.8; LCMS *m/z*: 321 (^{81}Br MH)$^+$.

2-(4-Bromophenyl)-5-((2-methoxyethyl)amino)oxazole-4-carbonitrile 5

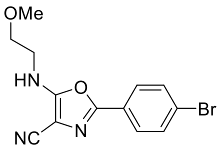

To a solution of **4** (1.00 g, 2.50 mmol) and triethylamine (0.87 mL, 5.00 mmol) in MeCN (40 mL) cooled to 0 °C, was added 2-methoxyethanolamine (0.81 mL, 7.50 mmol) and the mixture was left to stir overnight at room temperature. The volatiles were removed under reduced pressure, and the residue dissolved in EtOAc (40 mL), washed with water (4 × 40 mL), dried over $MgSO_4$, filtered and concentrated under reduced pressure to give the crude product, which was purified by column chromatography on silica (2:1 petroleum ether: EtOAc) to give **5** as a colourless solid (0.54 g, 67%); ^{1}H NMR (400 MHz, $CDCl_3$) δ 7.72 (2H, d, J = 8.8 Hz), 7.57 (2H, d, J = 8.8 Hz), 5.51 (1H, t, J = 5.2 Hz), 3.63–3.71 (4H, m), 3.44 (3H, s); ^{13}C NMR (101 MHz, $CDCl_3$) δ 160.8, 150.1, 132.2, 127.1, 125.1, 124.7, 115.1, 86.8, 70.3, 59.1, 43.5; ; LCMS *m/z*: 324 (^{81}Br MH)$^+$.

GSK1362 2-(4-((4-Chlorobenzyl)(methyl)amino)phenyl)-5-((2-methoxyethyl)amino)oxazole-4-carbonitrile 1

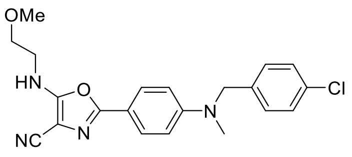

Aryl bromide **5** (0.065 g, 0.202 mmol) and 1-(4-chlorophenyl)-*N*-methylmethanamine (38 mg, 0.242 mmol) were added to a reaction flask under nitrogen, followed by anhydrous 1,4-dioxane (2.0 mL) and LiHMDS (1.0 M solution in THF, 0.71 mL, 0.71 mmol). To the solution, RuPhos ligand (9 mg, 0.02 mmol) and RuPhosPd precatalyst (16 mg, 0.02 mmol) were added and the mixture heated to 90 °C for 3 h. The mixture was loaded onto a C18 SPE cartridge (1.0 g, preconditioned with 2 mL MeCN), using additional MeOH (2.0 mL) to wash the crude product through. The volatiles were removed under reduced pressure to give the crude product, which was purified by automated reverse-phase column chromatography (MeCN/H_2O with formic acid modifier) to give **GSK1362** (**1**) as a light yellow solid (22 mg, 27%); ^{1}H NMR (400 MHz, $CDCl_3$) δ 7.69 (2H, d, J = 7.2 Hz), 7.29 (2H, d, J = 6.4 Hz), 7.13 (2H, d, J = 6.4 Hz), 6.70 (2H, d, J = 7.2 Hz), 5.14 (1H, t, J = 4.4 Hz), 4.56 (2H, s), 3.59–3.66 (4H, m), 3.41 (3H, s), 3.08 (3H, s); ^{13}C NMR (101 MHz, $CDCl_3$) δ 160.3, 152.6, 150.8, 136.6, 133.0, 129.0, 128.0, 127.4, 115.7, 114.5, 112.0, 86.3, 70.5, 59.1, 55.7, 43.6, 38.8; LCMS *m/z*: 397 (MH)$^+$.

Supplemental figures and tables

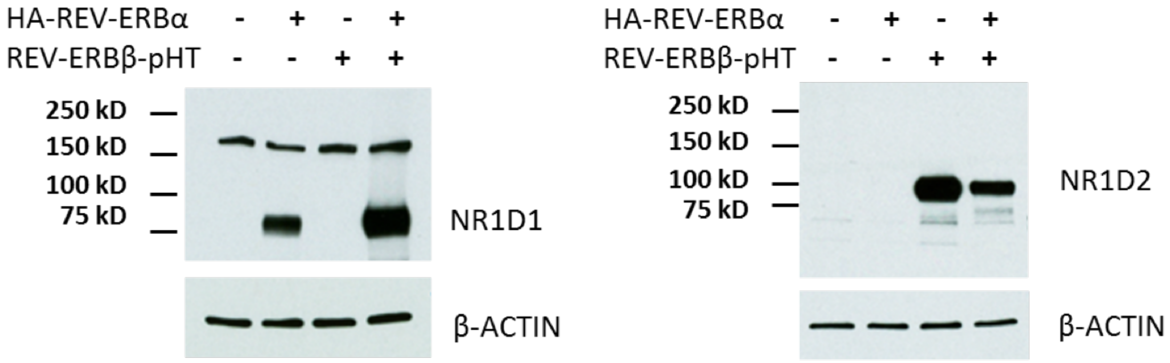

Supplemental Figure 1: **Specificity of antibody GSK6F05 for REV-ERBα**

HEK 293 cells were transfected with HA-REV-ERBα and REV-ERBβ-Halotag. Total cell lysates were analysed by Western blotting for REV-ERBα protein levels using mouse monoclonal antibody GSK6F05 and for REV-ERBβ protein levels using rabbit polyclonal antibody (Proteintech, # 13906-1-AP). Non specific band at about 150 kDa detected with the GSK6F05 antibody.

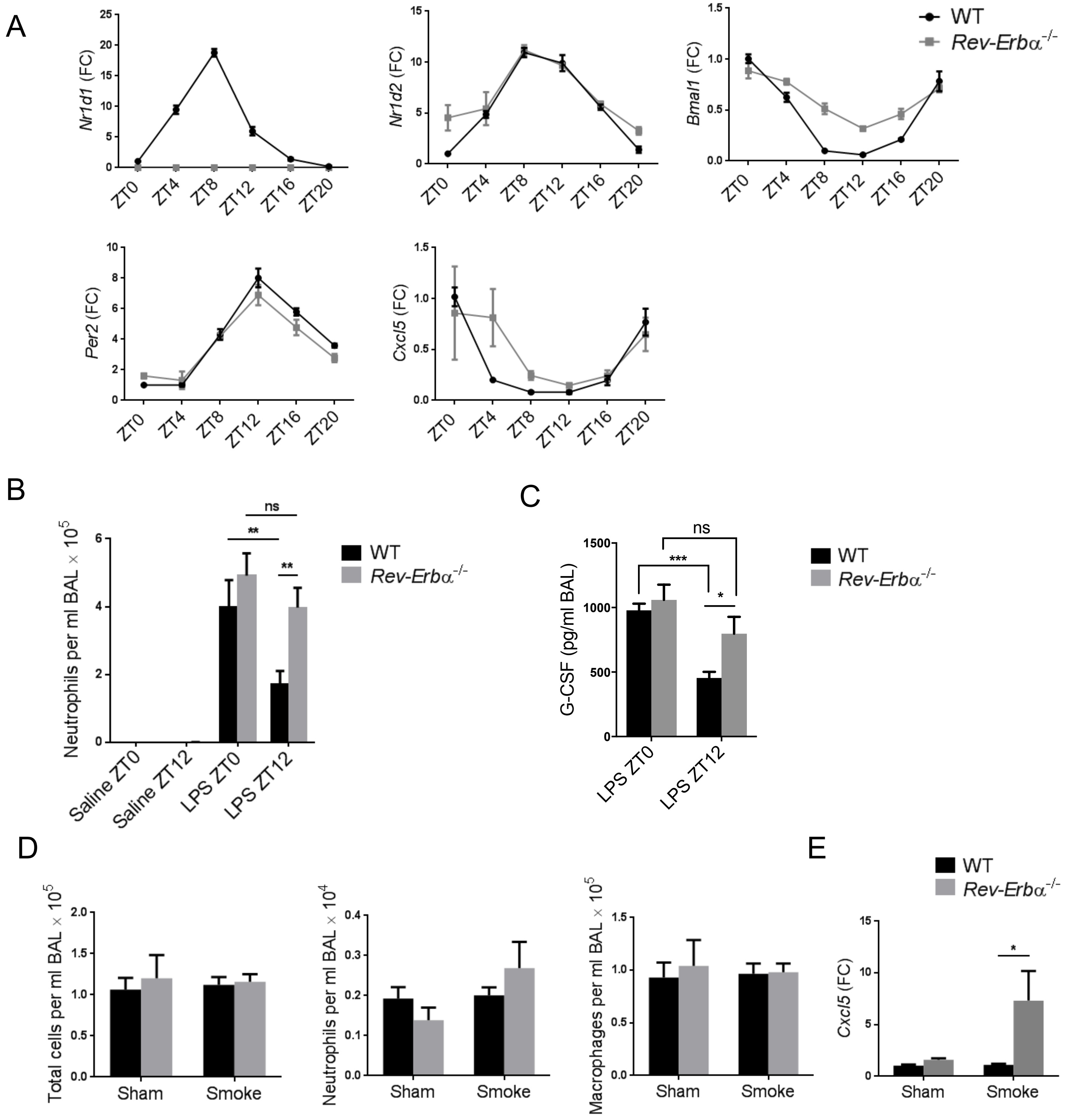

Supplemental Figure 2: *Rev-Erbα*$^{-/-}$ mice possess an intact clock within the lung but exhibit exaggerated pulmonary innate immune responses

(A) Q-PCR analysis in whole lung tissues. Data normalized to WT at ZT0 and presented as mean ± SEM; *n*=5-6 for WT and *n*=3-5 for *Rev-Erbα*$^{-/-}$ per time point.

(B,C) *Rev-Erbα*$^{-/-}$ and WT mice were exposed to aerosolised LPS (2 mg/ml) at ZT0 or ZT12 and culled 5 hours later. (B) Neutrophil numbers in BAL samples were determined by flow cytometry. (C) G-CSF protein levels in BAL samples, measured using multiplex assay. Data presented as mean ± SEM; *n*=6-10*,* *P <0.05, **P <0.01, ***P <0.001 (Two-way ANOVA, post hoc Bonferroni).

(D-E) Single cigarette smoke exposures was performed between ZT8 and ZT10 and animals were culled 24 hours after exposure. (D) Cell numbers in BAL samples. Data presented as mean ± SEM, *n* = 9-10. (E) Q-PCR analysis of *Cxcl5* in whole lung. Data normalized to sham wild-type group and presented as mean ± SEM; *n* = 9-10, *P <0.05 (Two-way ANOVA, post hoc Bonferroni).

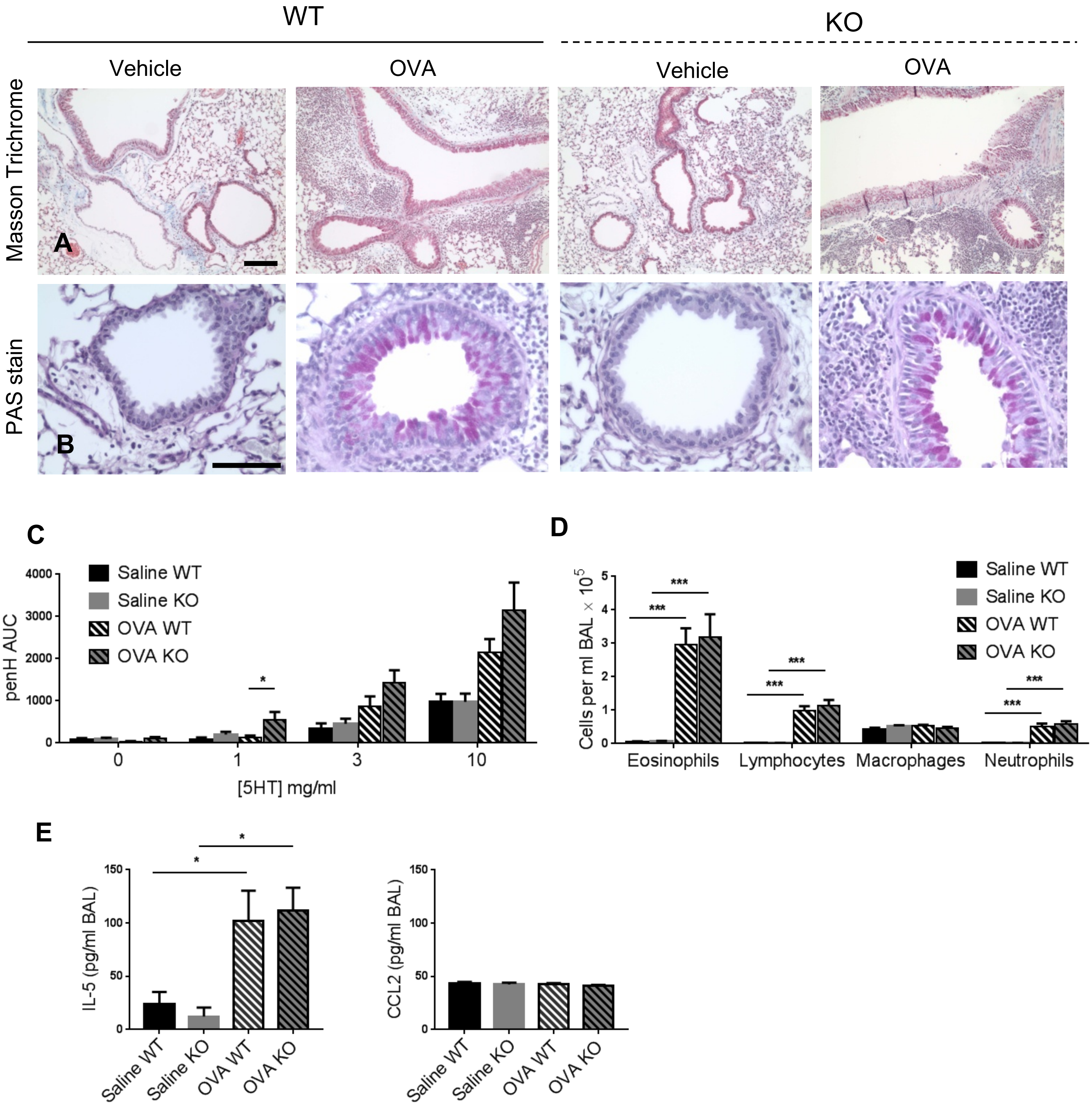

Supplemental Figure 3: *Rev-Erbα*$^{-/-}$ mice exhibit similar responses to OVA challenge than littermate controls

(A) Masson trichrome staining showing extensive inflammatory cell invasion after OVA challenge (collagen blue, cell nuclei purple).

(B) PAS staining showing mucus production by airway goblet cells after OVA challenge (mucus pink, cell nuclei purple) scale bars = 50µm.

(C) Airway hyper-responsiveness to 5HT assessed using non-invasive lung function. Data represented as mean ± SEM, *n*=7-12 (One way ANOVA, Bonferroni).

(D) Cell numbers in BAL samples after OVA challenge. Data represented as mean ± SEM, *n*=7-12 (One way ANOVA, Bonferroni).

(E) IL-5 and CCL2 levels in the same BAL samples. Data represented as mean ± SEM, *n*=7-12 (One way ANOVA, Bonferroni).

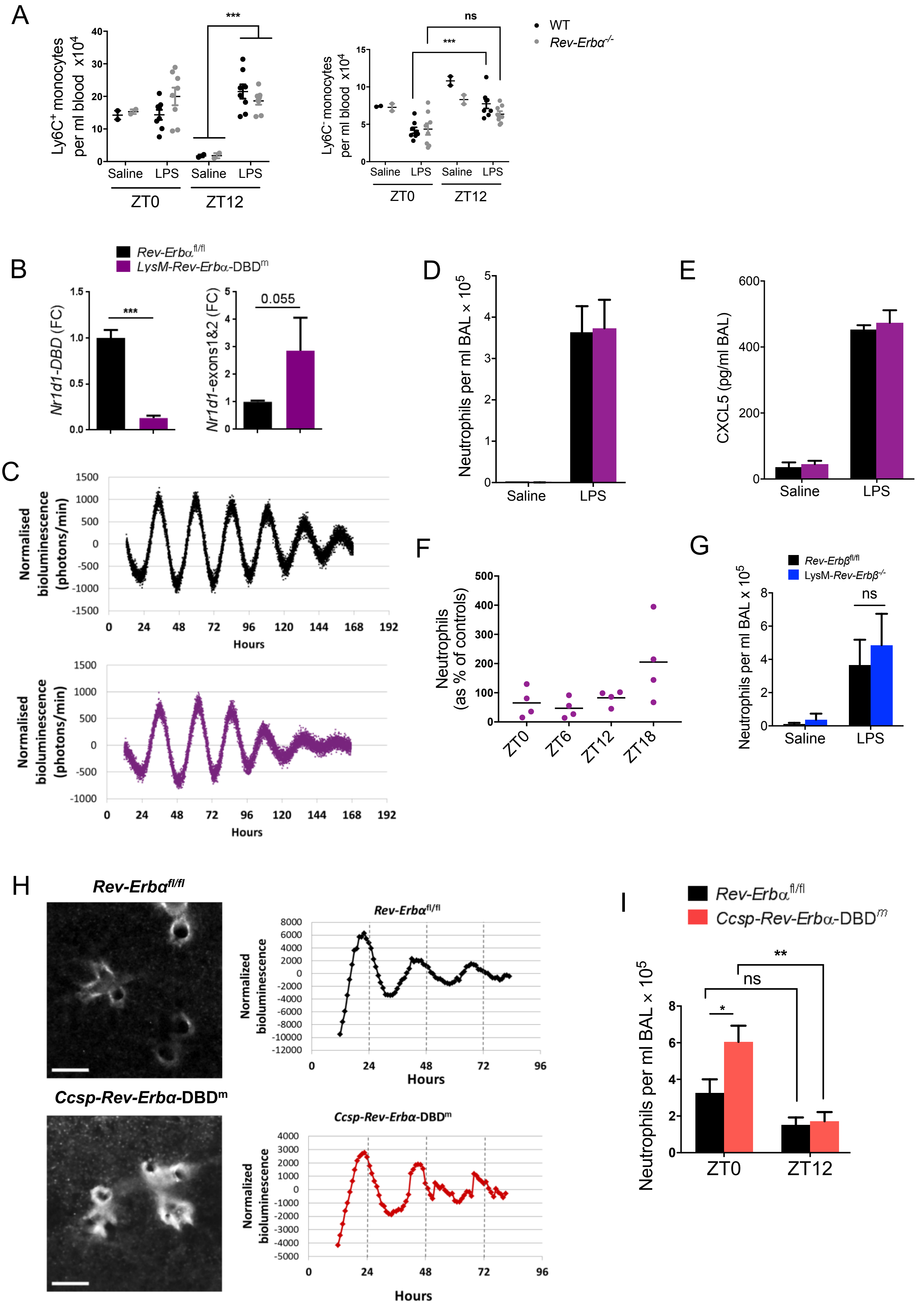
A
Ly6C+ monocytes per ml blood x10^4
Saline LPS Saline LPS
ZT0 ZT12
***
Ly6C- monocytes per ml blood x10^4
ns
***
WT
Rev-Erbα-/-
B
Rev-Erbα fl/fl
LysM-Rev-Erbα-DBDm
Nr1d1-DBD (FC)
Nr1d1-exons1&2 (FC)
0.055
C
Normalised bioluminescence (photons/min)
Hours
D
Neutrophils per ml BAL × 10^5
Saline LPS
E
CXCL5 (pg/ml BAL)
Saline LPS
F
Neutrophils (as % of controls)
ZT0 ZT6 ZT12 ZT18
G
Rev-Erbβ fl/fl
LysM-Rev-Erbβ-/-
Neutrophils per ml BAL x 10^5
ns
Saline LPS
H
Rev-Erbα fl/fl
Ccsp-Rev-Erbα-DBDm
Normalized bioluminescence
Hours
I
Rev-Erbα fl/fl
Ccsp-Rev-Erbα-DBDm
Neutrophils per ml BAL × 10^5
ns
**
*
ZT0 ZT12

Supplemental Figure 4: Effect of impaired REV-ERBα or REV-ERBβ in myeloid or airway epithelial cell lineage on pulmonary inflammation

(A) Bone marrow cells from *Rev-Erbα*$^{-/-}$ or littermate controls were transplanted into wild-type recipient mice which were then exposed to aerosolised LPS at 2 mg/ml or saline at indicated times for 20 min. Monocytes in blood samples collected 5 hours after challenge were determined by flow cytometry analyses. Data presented as mean ± SEM; n = 2 (saline) or 5-8 (LPS), ***P <0.001 (Two-way ANOVA, post hoc Bonferroni).

(B) Q-PCR analysis in PECs collected at ZT9, seeded into plates and let to attach for 2 hours before lysis. Data normalized to *Rev-Erbα*$^{fl/fl}$ littermate control group and presented as mean ± SD; *n* = 3. ***P <0.001 (Student's *t*-test).

(C) PER2 bioluminescence recordings from *Rev-Erbα*$^{fl/fl}$ (black) and *LysM-Rev-Erbα*-DBD^{m} (purple) PECs. Photon counts per minute were normalised to a 24 hour moving average and traces are representative of 3 biological replicates.

(D,E) *LysM-Rev-Erbα*-DBD^{m} and littermate control mice were exposed to aerosolised LPS at 2 mg/ml or saline at ZT4 for 20 min. (D) Neutrophil numbers in the BAL samples collected 5 hours after challenge were determined by flow cytometry analyses. (E) CXCL5 protein levels in BAL samples were assessed by multiplex assay. Data presented as mean ± SEM; *n* = 7-9 (Two-way ANOVA, post hoc Bonferroni).

(F) *LysM-Rev-Erbα*-DBD^{m} and littermate control mice were exposed to aerosolised LPS at 2 mg/ml at indicated times for 20 min. Neutrophil numbers in the BAL samples collected 5 hours after challenge were determined by flow cytometry analyses. Data presented as percentage of littermate control neutrophil numbers; *n* = 4 per time point.

(G) *LysM-Rev-Erbβ*$^{-/-}$ and littermate control mice were exposed to aerosolised LPS at 2 mg/ml or saline at ZT4 for 20 min. Neutrophil numbers in the BAL samples collected 5 hours after challenge were determined by flow cytometry analyses. Data presented as mean ± SEM; *n* = 8 (Two-way ANOVA, post hoc Bonferroni).

(H) Snapshots of PER2 oscillations in bronchioles within precision cut lung slices. Scale bars, 500 µM. Bioluminescence intensity from bronchioles was quantified, normalised to a 24 hour moving average. Traces are representative of 2 biological replicates.

(I) *Ccsp-Rev-Erbα*-DBD^{m} and littermate control mice were exposed to aerosolised LPS at 2 mg/ml or saline at ZT0 or ZT12 for 20 min. Neutrophil numbers in the BAL samples collected at ZT5 or ZT17 were determined by flow cytometry analyses. Data presented as mean ± SEM; *n* = 16 at ZT0 and *n* = 7-8 at ZT12, *P <0.05, **P <0.01 (Two-way ANOVA, post hoc Bonferroni).

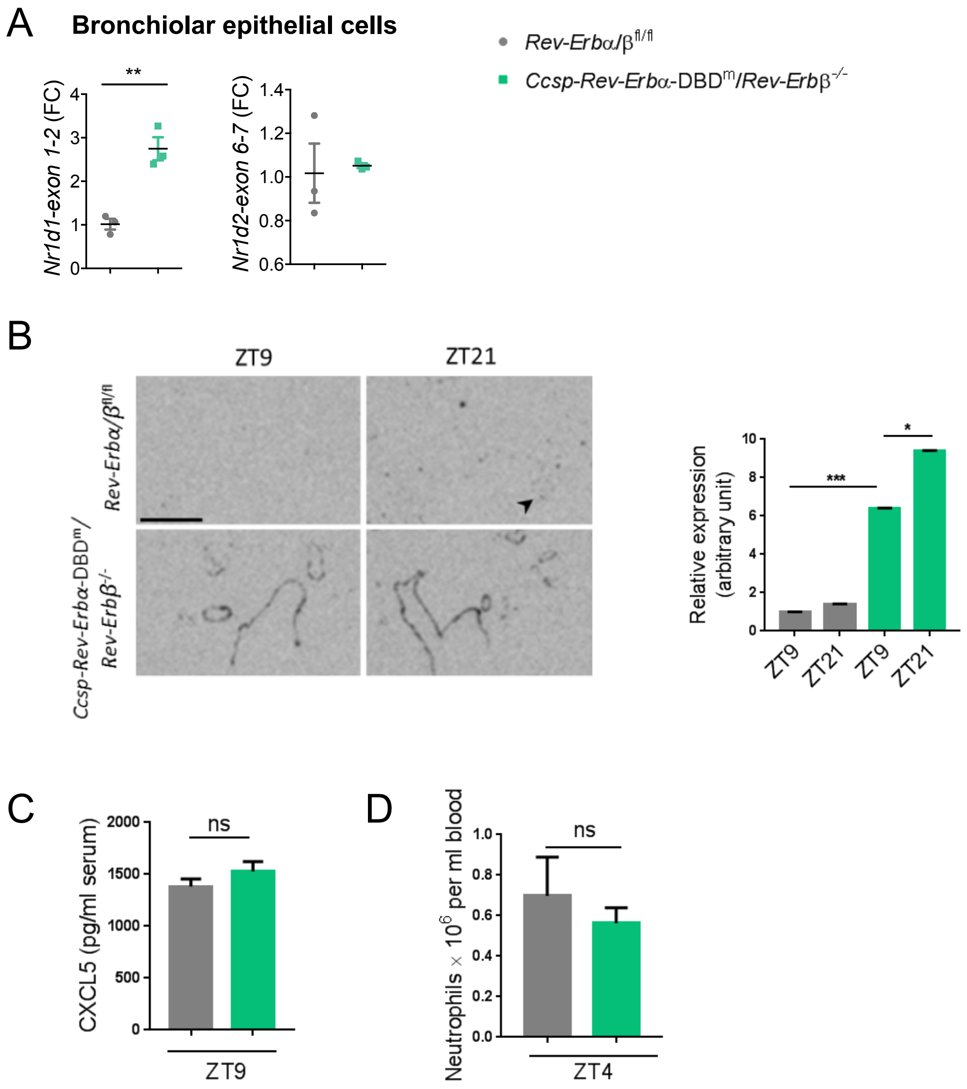

Supplemental Figure 5: Effect of impaired REV-ERBα DBD and REV-ERBβ deletion in CCSP-expressing cells

(A) Q-PCR analysis of DBD or exon 4-truncated transcripts of *Rev-Erbα* and *Rev-Erbβ* respectively in bronchial epithelial cells, laser captured from lung tissues collected at ZT9. Data normalized to *Rev-Erbα/β$^{fl/fl}$* control group and presented as mean ± SD; n = 3.

(B) Example images of lung slices probed *Cxcl5* mRNA via *in situ* hybridisation (scale bar = 5mm) and relative quantification of mRNA abundance from multiple lung slices. Data presented as mean ± SEM; *n* = 2/genotype/time point. *P <0.05, ***P <0.001 (One-way ANOVA, post hoc Bonferroni).

(C) CXCL5 protein levels in serum samples were assessed by multiplex assay. Data presented as mean ± SEM; *n* = 7/genotype, Student's t test.

(D) Neutrophil numbers in blood were determined by flow cytometry analysis. Data presented as mean ± SEM; *n* = 5/genotype, Student's t test.

A

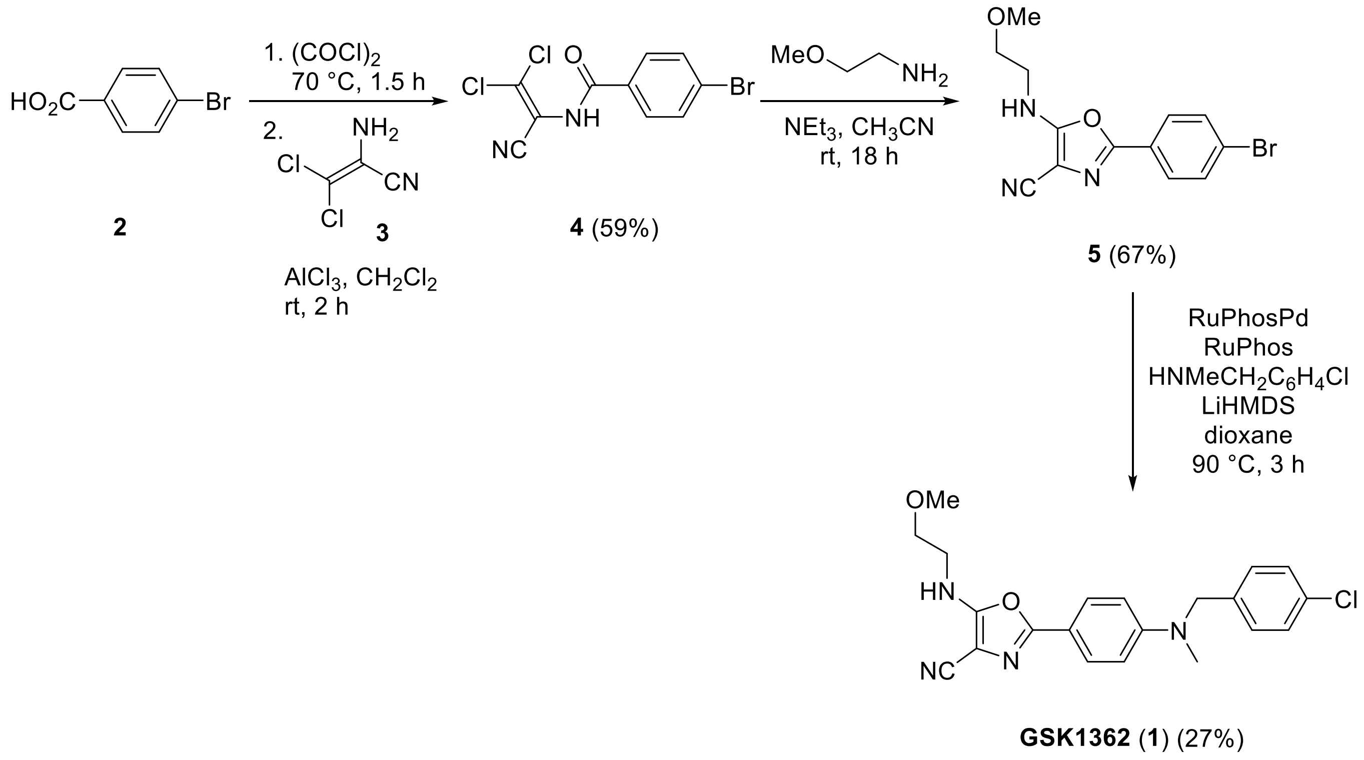

B

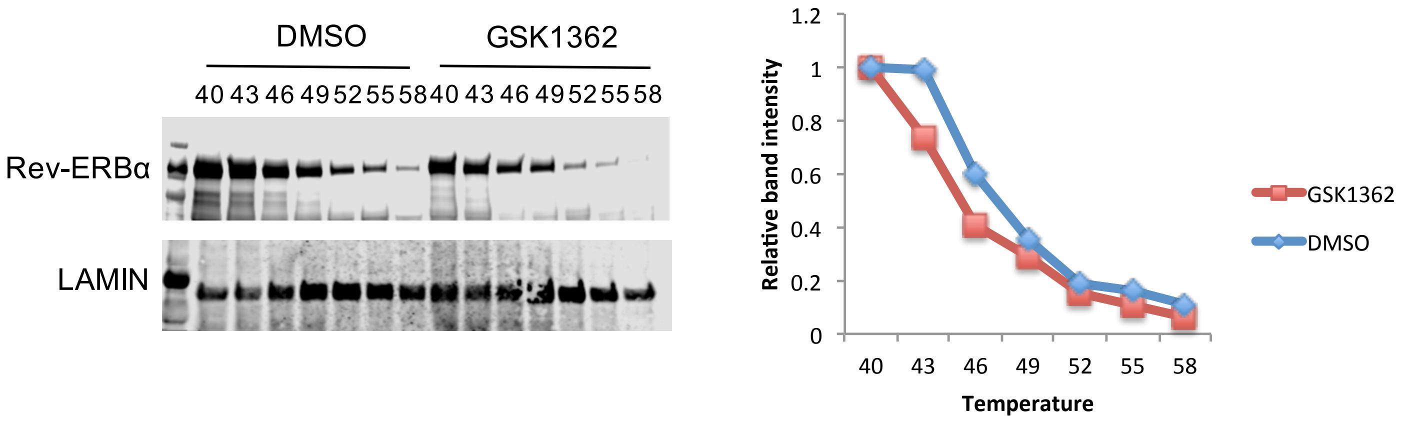

Supplemental Figure 6: Experimental procedures for preparation of GSK1362 and its effect on REV-ERBα using cellular thermal shift assay

(A) GSK1362 (**1**) can be prepared in 4-steps and 11% overall yield from commercial building blocks. Full details for the synthesis of **1** are provided in the supplemental experimental procedures.

(B) HEK293T cells were transfected with Halotag Rev-Erbα and treated with GSK1362 at 10 μM for 1 hour. Aliquots of harvested cells (without washing) were heated for 3 min at each temperature, frozen and lysed with 0.2% triton. REV-ERBα was imaged, and quantified using a halo-ligand (Alexa660). Representative of *n*=3.

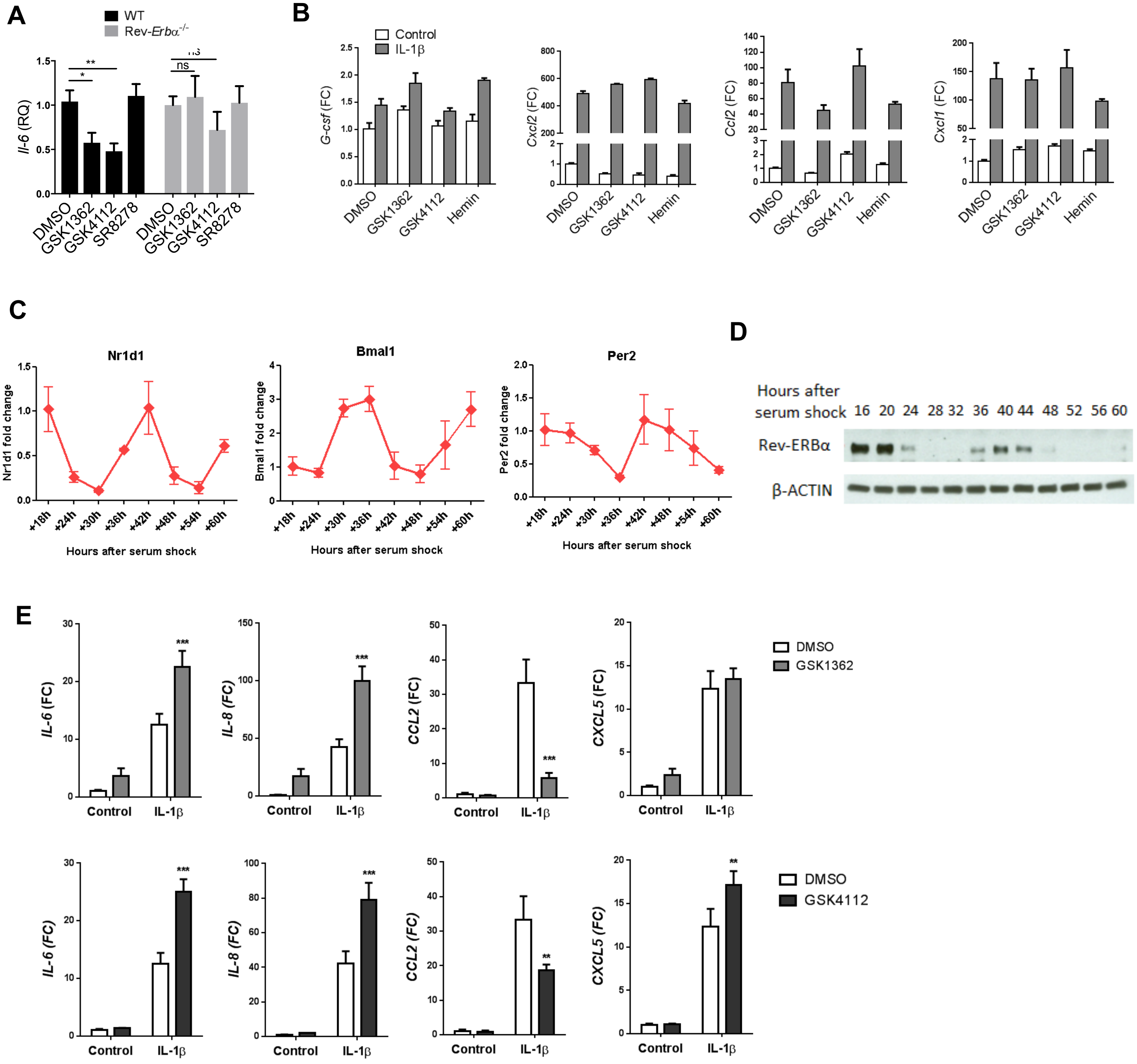

Supplemental Figure 7: Effects of GSK1362 in PECS, BMDMs and bronchial epithelial cells
(A) BMDMs from *Rev-Erbα*-/- mice or littermate controls were treated with compounds at 10 µM in presence of LPS at 100 ng/ml for 4 hours. Gene expression was determined by Real-Time qPCR and normalized to un-treated control cells. Data are presented as mean ± SD; n = 3. *P<0.05, **P <0.01, ***P <0.001 (One-way ANOVA, post hoc Bonferroni).
(B) Q-PCR analysis of transcript levels in LA-4 cells synchronised by serum shock, treated 16 hours with ligands at 10 µM, followed 2 hours later by IL-1β at 1 ng/ml for additional 2 hours. Data normalized to un-stimulated control cells and presented as mean ± SD, representative of *n*=3.
(C) Q-PCR analysis of transcript levels in NHBE cells synchronised by serum shock and lysed every 6 hours over 42 hours. Data normalized to the first time point and presented as mean ± SD; *n*=3.
(D) REV-ERBα protein levels in in NHBE cells synchronised by serum shock whole lungs and lysed every 4 hours over 44 hours. Representative of *n*=3.
(E) Q-PCR analysis of transcript levels in NHBE cells synchronised by serum shock, treated 16 hours with ligands at 10 µM, followed 2 hours later by IL-1β at 1 ng/ml for additional 2 hours. Data normalized to un-stimulated control cells and presented as mean ± SD, representative of *n*=3 (Two-way ANOVA, post hoc Bonferroni).

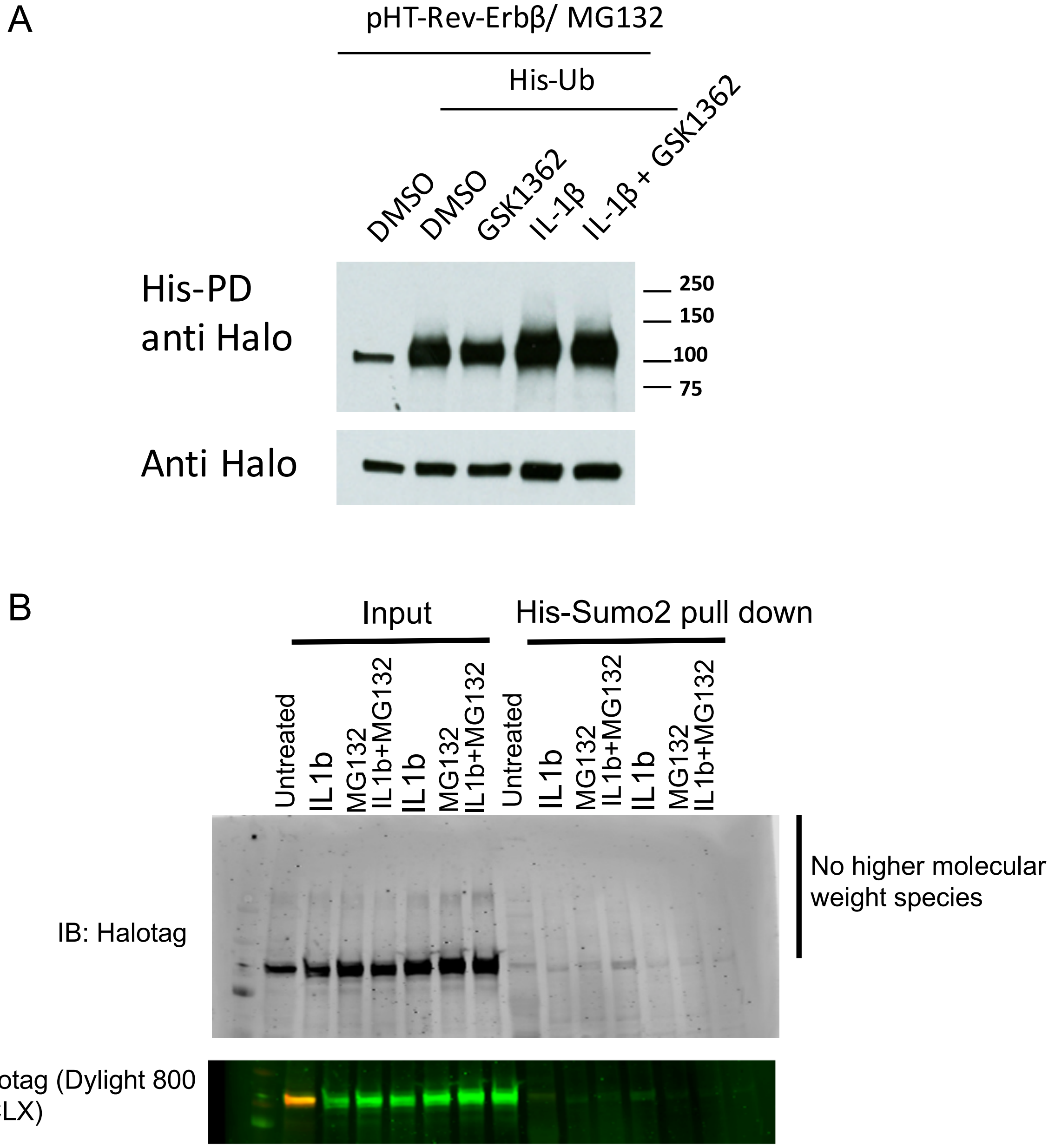

Supplemental Figure 8: REV-ERBβ post-translational modifications

(A) Ubiquitinated REV-ERBβ protein in HEK293T cells transfected with HaloTag-Rev-Erbβ and His-Ub plasmids, and treated with GSK1362 at 10 μM and IL-1β at 5 ng/ml for 4 hours in the presence of MG132 at 5 μM. Representative of *n*=3.

(B) SUMO2 ligation to REV-ERBβ protein in HEK293T cells transfected with HaloTag-Rev-Erbβ, His-SUMO2 and Ubc9 plasmids, and treated with GSK1362 at 10 μM, IL-1β at 5 ng/ml, MG132 at 5 μM for 4 hours. Untreated also contains Halotag ligand Alexa660, confirming identification of Reverbβ (turns yellow). Representative of *n*=3.

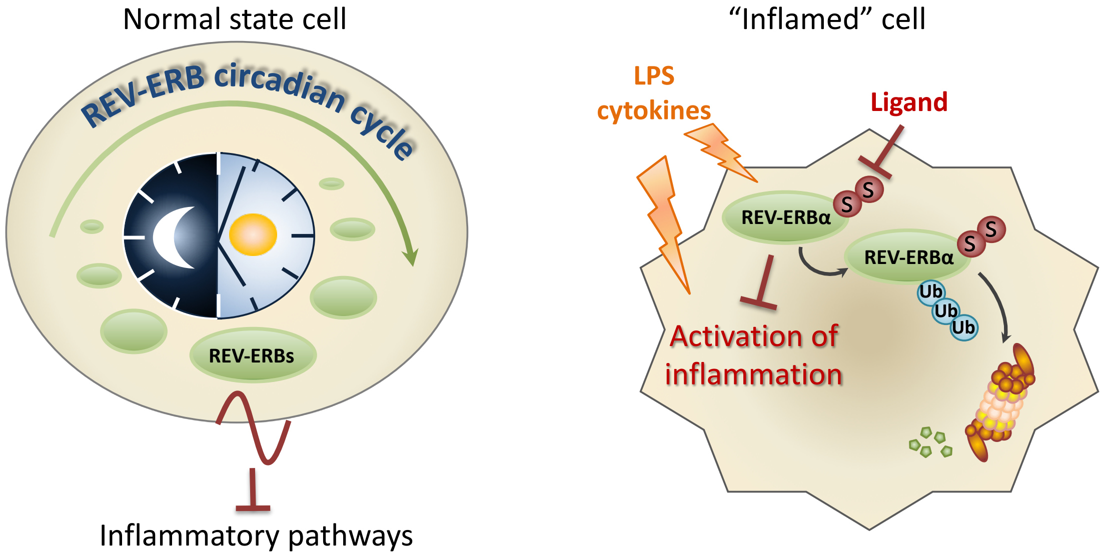

Supplemental Figure 9: Schematic representation of a new homeostatic circuit linking inflammation and REV-ERBα

Under resting, non-stress conditions, REV-ERBα and its paralog REV-ERBβ act as rhythmic repressors to limit inflammatory activity. Upon inflammatory triggers, such as LPS or cytokine challenges, REV-ERBα is SUMOylated, which drives the protein ubiquitination, allowing the proteosomal degradation of a repressor and therefore development of a full immune response. Treatment with synthetic REV-ERBα ligand GSK1362 blocks this degradation pathway, suggesting potential pharmacological intervention to enhance activity of an inhibitor of inflammation in a context of chronic inflammatory diseases.

Cytokine/chemokine	wildtype	*Rev-Erbα*$^{-/-}$	significance
IL1a	20.1 ± 2.7	25.1 ± 3.84	ns
IL1b	226.5 ± 20.5	339.7 ± 56.7	ns
IL2	8.0 ± 0.5	9.1 ± 0.8	ns
IL3	1.3 ± 0.2	1.8 ± 0.2	ns
IL5	1.6 ± 0.2	2.9 ± 0.3	0.005
IL6	794.2 ± 56.3	1638.0 ± 659.0	ns
IL9	51.5 ± 3.5	78.5 ±1 4.3	ns
IL10	10.7 ± 0.7	17.9 ± 4.4	ns
IL12 (P40)	601.6 ± 98.3	426.7 ± 34.8	ns
IL13	310.8 ± 8.6	527.4 ± 199.5	ns
IL17	5.1 ± 0.8	8.4 ± 0.8	0.012
GCSF	4566.9 ± 739.8	8835.5 ± 616.3	***
GMCSF	399.7 ± 50.8	363.2 ± 92.7	ns
IFN γ	33.8 ± 5.1	41.5 ± 3.5	ns
CXCL1 (KC)	239.4 ± 34.1	1429.5 ± 346.0	*
CXCL2	647.1 ± 61.4	3374.5 ± 578.0	**
CXCL5	1212 ± 263	4927 ± 543	***
CXCL10 (IP10)	483.7 ± 49.5	529.2 ± 66.5	ns
CCL2 (MCP1)	102.8 ± 9.0	275.1 ± 55.4	*
CCL3 (MIP1α)	6044.4 ± 1010.0	12666.7 ± 3013.5	ns (0.09)
CCL4 (MIP1 β)	169.2 ± 18.9	864.8 ± 222.7	*
CCL5 (RANTES)	189.5 ± 17.6	181.3 ± 27.1	ns
CCL11 (EOTAXIN)	474.5 ± 43.8	745.9 ± 44.5	***
TNF α	502.3 ± 133.6	2601.6 ± 838.5	*
M-CSF	1.8 ± 0.1	1.6 ± 0.3	ns

Supplemental Table 1: cytokine/chemokine levels in BAL from wild-type and *Rev-Erbα*$^{-/-}$ mice exposed to LPS at ZT4

Mice were exposed to aerosolised LPS (2mg/ml) at ZT4 for 20 minutes and culled 5 hours later. Cytokine/chemokine levels were measured using magnetic luminex assay. Values are presented as mean ± SEM, n=8, Student's t test.

	ZT0			ZT12		
Cytokine/Chemokine	WT	Rev-Erbα$^{-/-}$	Significance	WT	Rev-Erbα$^{-/-}$	Significance
IL-1α	OOR <	OOR <		OOR <	OOR <	
IL-1β	OOR <	OOR <		OOR <	OOR <	
IL-2	OOR <	OOR <		OOR <	OOR <	
IL-5	OOR <	OOR <		OOR <	OOR <	
IL-6	747.7 ± 45.18	735.9 ± 61.01	ns	598.2 ± 48.75	644.7 ± 86.64	ns
IL-10	OOR <	OOR <		OOR <	OOR <	
IL-12p70	OOR <	OOR <		OOR <	OOR <	
IL-13	OOR <	OOR <		OOR <	OOR <	
IL-17	OOR <	OOR <		OOR <	OOR <	
G-CSF	1001 ± 62.14	1017 ± 90.82	ns	454.2 ± 47.65	797.8 ± 130.3	*
GM-CSF	70.24 ± 11.18	84.96 ± 15.14	ns	83.53 ± 10.80	103.4 ± 19.78	ns
IFN-γ	OOR <	OOR <		OOR <	OOR <	
CXCL1 (KC)	646.1 ± 50.70	870.7 ± 105.8	ns	655.0 ± 44.63	1701 ± 566.4	*
CXCL2	670.4 ± 48.06	1662 ± 381.5	ns	640.7 ± 50.94	2206 ± 654.8	*
CXCL5	626.8 ± 23.10	784.2 ± 33.54	***	677.8 ± 29.92	668.3 ± 19.22	ns
CXCL10 (IP10)	210.5 ± 36.92	186.4 ± 24.90	ns	187.2 ± 15.85	240.6 ± 63.20	ns
CCL2 (MCP-1)	1534 ± 66.83	1352 ± 58.11	ns	1524 ± 62.94	1383 ± 115.7	ns
CCL3 (MIP-1α)	2402 ± 238.6	1811 ± 178.3	ns	2298 ± 165.4	1647 ± 203.3	ns
CCL4 (MIP-1β)	9226 ± 682.7	7153 ± 557.1	ns	9434 ± 720.3	7648 ± 1088	ns
CCL5 (RANTES)	390.8 ± 39.37	295.5 ± 18.86	ns	453.4 ± 33.50	340.7 ± 36.24	*
CCL11 (EOTAXIN)	OOR <	OOR <		OOR <	OOR <	
TNF-α	113.7 ± 6.188	101.8 ± 7.588	ns	84.01 ± 5.339	75.62 ± 9.763	ns
M-CSF	OOR <	OOR <		OOR <	OOR <	

Supplemental Table 2: cytokine/chemokine levels in BAL from wild-type and *Rev-Erbα$^{-/-}$* mice exposed to LPS at ZT0 or ZT12

Mice were exposed to aerosolised LPS (2mg/ml) at ZT0 or ZT12 for 20 minutes and culled 5 hours later. Cytokine/chemokine levels were measured using magnetic luminex assay. OOR< = Out of Range Below.

Values are presented as mean ± SEM, *n*=6-10, Student's t test.

Cytokine/Chemokine	*Rev-Erbα*$^{fl/fl}$	*Ccsp-Rev-Erbα-DBD*^{m}	Significance
IL-1α	*OOR<*	*OOR<*	
IL-1β	*OOR<*	*OOR<*	
IL-2	*OOR<*	*OOR<*	
IL-5	*OOR<*	*OOR<*	
IL-6	*859.7 ± 95.86*	*731.7 ± 104.8*	ns
IL-10	*OOR<*	*OOR<*	
IL-12p70	*OOR<*	*OOR<*	
IL-13	*OOR<*	*OOR<*	
IL-17	*OOR<*	*OOR<*	
G-CSF	*923.9 ± 40.57*	*915.0 ± 89.11*	ns
GM-CSF	*75.35 ± 8.890*	*47.92 ± 8.625*	*
IFN-γ	*OOR<*	*OOR<*	
CXCL1 (KC)	*232.3 ± 35.96*	*202.7 ± 25.14*	ns
CXCL2	*415.5 ± 53.11*	*324.1 ± 22.67*	ns
CXCL5	*422.5 ± 34.40*	*705.4 ± 35.26*	***
CXCL10 (IP10)	*1031 ± 172.1*	*475.5 ± 95.55*	*
CCL2 (MCP-1)	*3030 ± 397.9*	*2683 ± 372.6*	ns
CCL3 (MIP-1α)	*3080 ± 553.9*	*1691 ± 280.0*	ns (0.0521)
CCL4 (MIP-1β)	*7653 ± 1714*	*5615 ± 1340*	ns
CCL5 (RANTES)	*445.7 ± 92.24*	*402.8 ± 91.48*	ns
CCL11 (EOTAXIN)	*25.79 ± 6.811*	*17.73 ± 5.736*	ns
TNF-α	*104.2 ± 13.94*	*113.8 ± 19.25*	ns
M-CSF	*19.17 ± 1.976*	*14.04 ± 1.726*	ns

Supplemental Table 3: cytokine/chemokine levels in BAL from *Rev-Erbα*$^{fl/fl}$ and *Ccsp-Rev-Erbα-DBD*^{m} mice exposed to LPS at ZT4

Mice were exposed to aerosolised LPS (2mg/ml) at ZT4 for 20 minutes and culled 5 hours later. Cytokine/chemokine levels were measured using magnetic luminex assay. OOR< = Out of Range Below. Values are presented as mean ± SEM, *n*=5-9, Student's t test.

	ZT0			ZT12		
Cytokine/Chemokine	*Rev-Erbα*$^{fl/fl}$	*Ccsp-Rev-Erbα*-DBD^{m}	Significance	*Rev-Erbα*$^{fl/fl}$	*Ccsp-Rev-Erbα*-DBD^{m}	Significance
IL-1α	OOR <	OOR <		OOR <	OOR <	
IL-1β	OOR <	OOR <		OOR <	OOR <	
IL-2	OOR <	OOR <		OOR <	OOR <	
IL-5	OOR <	OOR <		OOR <	OOR <	
IL-6	659.9 ± 102.9	713.8 ± 101.8	ns	250.2 ± 59.45	175.0 ± 45.36	ns
IL-10	OOR <	OOR <		OOR <	OOR <	
IL-12p70	OOR <	OOR <		OOR <	OOR <	
IL-13	OOR <	OOR <		OOR <	OOR <	
IL-17	OOR <	OOR <		OOR <	OOR <	
G-CSF	975.0 ± 156.3	1267 ± 162.0	ns	305.3 ± 30.56	294.9 ± 47.99	ns
GM-CSF	24.58 ± 4.480	19.62 ± 6.950	ns	45.60 ± 12.16	31.90 ± 6.553	ns
IFN-γ	OOR <	OOR <		OOR <	OOR <	
CXCL1 (KC)	528.3 ± 79.01	487.8 ± 46.29	ns	644.4 ± 74.42	500.8 ± 37.63	ns
CXCL2	622.6 ± 99.43	641.9 ± 74.34	ns	575.7 ± 102.6	408.6 ± 80.62	ns
CXCL5	664.4 ± 39.24	886.2 ± 63.18	*	801.3 ± 99.89	755.4 ± 46.42	ns
CXCL10 (IP10)	OOR <	OOR <		OOR <	OOR <	
CCL2 (MCP-1)	1799 ± 382.5	2180 ± 307.2	ns	946.8 ± 300.7	573.6 ± 213.9	ns
CCL3 (MIP-1α)	1348 ± 248.9	1253 ± 264.2	ns	1002 ± 213.1	1081 ± 210.4	ns
CCL4 (MIP-1β)	2811 ± 792.5	4403 ± 848.4	ns	1738 ± 718.0	772.9 ± 381.7	ns
CCL5 (RANTES)	271.5 ± 77.78	342.8 ± 66.85	ns	156.75 ± 25.77	OOR <	
CCL11 (EOTAXIN)	OOR <	OOR <		OOR <	OOR <	
TNF-α	87.69 ± 14.74	115.6 ± 12.68	ns	33.00 ± 6.348	30.22 ± 7.513	ns
M-CSF	OOR <	OOR <		OOR <	OOR <	

Supplemental Table 4: cytokine/chemokine levels in BAL from *Rev-Erbα*$^{fl/fl}$ and *Ccsp-Rev-Erbα-DBD*^{m} mice exposed to LPS at ZT0 or ZT12

Mice were exposed to aerosolised LPS (2mg/ml) at ZT0 or ZT12 for 20 minutes and culled 5 hours later. Cytokine/chemokine levels were measured using magnetic luminex assay. OOR< = Out of Range Below.

Values are presented as mean ± SEM, *n*=7-9, Student's t test.

Cytokine/Chemokine	*Rev-Erbα$^{fl/fl}$*	*Ccsp-Rev-Erbα-DBD^{m}*	Significance	*Rev-Erba/β$^{fl/fl}$*	*Ccsp-Rev-Erbα-DBD^{m}/Rev-Erbβ$^{-/-}$*	Significance
IL-1α	*45.83 ± 5.291*	*39.12 ± 4.465*	ns	*68.65 ± 9.544*	*83.90 ± 12.60*	ns
IL-1β	OOR <	OOR <		OOR <	OOR <	
IL-2	OOR <	OOR <		*OOR <*	*OOR <*	
IL-5	OOR <	OOR <		OOR <	OOR <	
IL-6	*487.1 ± 131.8*	*672.4 ± 119.9*	ns	*1862 ± 194.3*	*1110 ± 82.62*	**
IL-10	OOR <	OOR <		*OOR <*	*OOR <*	
IL-12p70	OOR <	OOR <		*OOR <*	*OOR <*	
IL-13	OOR <	OOR <		*OOR <*	*OOR <*	
IL-17	OOR <	OOR <		*OOR <*	*OOR <*	
G-CSF	*426.0 ± 85.57*	*717.9 ± 151.2*	ns	*1309 ± 177.9*	*2031 ± 108*	**
GM-CSF	*37.76 ± 8.484*	*27.46 ± 4.782*	ns	*95.09 ± 12.62*	*40.14 ± 9.888*	**
IFN-γ	OOR <	OOR <		*OOR <*	*OOR <*	
CXCL1 (KC)	*385.7 ± 79.86*	*541.6 ± 48.15*	ns	*874.3 ± 108.9*	*3353 ± 414.6*	****
CXCL2	*398.4 ± 82.25*	*571.7 ± 64.50*	ns	*953.0 ± 140.6*	*2240 ± 197.9*	****
CXCL5	*450.5 ± 80.58*	*874.1 ± 124.1*	****	*556.1 ± 37.05*	*1812 ± 39.65*	****
CXCL10 (IP10)	OOR <	OOR <		*291.1 ± 51.17*	*235.0 ± 32.55*	ns
CCL2 (MCP-1)	*728.3 ± 233.2*	*987.1 ± 374.1*	ns	*3169 ± 156.5*	*3589 ± 214.3*	ns
CCL3 (MIP-1α)	*1295 ± 369.7*	*913.8 ± 254.6*	ns	*2075 ± 220.2*	*2069 ± 315.4*	ns
CCL4 (MIP-1β)	*1081 ± 417.0*	*1659 ± 890.1*	ns	*7340 ± 551.1*	*9051 ± 1008*	ns
CCL5 (RANTES)	*93.42 ± 28.78*	*208.2 ± 106.1*	ns	*505.5 ± 51.63*	*265.1 ± 37.57*	*
CCL11 (EOTAXIN)	OOR <	OOR <		*12.99 ± 1.479*	*27.12 ± 3.572*	**
TNF-α	*26.18 ± 6.645*	*47.59 ± 13.63*	ns	*107.9 ± 11.25*	*131.1 ± 9.459*	ns
M-CSF	OOR <	OOR <		*OOR <*	*OOR <*	

Supplemental Table 5: cytokine/chemokine levels in BAL from *Rev-Erbα$^{fl/fl}$*, *Ccsp-Rev-Erbα-DBD^{m}*, *Rev-Erbα/β$^{fl/fl}$ and Ccsp-Rev-Erbα-DBD^{m}/Rev-Erbβ$^{-/-}$* mice exposed to LPS at ZT4

Mice were exposed to aerosolised LPS (2mg/ml) at ZT4 for 20 minutes and culled 5 hours later. Cytokine/chemokine levels were measured using magnetic luminex assay. OOR< = Out of Range Below.

Values are presented as mean ± SEM, *n*=5-9, Student's t test.

	ZT0			ZT12		
Cytokine/Chemokine	*Rev-Erbα/β$^{fl/fl}$*	*Ccsp-Rev-Erbα-DBDm-Rev-Erbβ$^{-/-}$*	Significance	*Rev-Erbα/β$^{fl/fl}$*	*Ccsp-Rev-Erbα-DBDm-Rev-Erbβ$^{-/-}$*	Significance
IL-1α	*30.47 ± 4.859*	*34.76 ± 7.309*	ns	*62.00 ± 4.557*	*49.54 ± 5.587*	ns
IL-1β	OOR <	OOR <		OOR <	OOR <	
IL-2	OOR <	OOR <		OOR <	OOR <	
IL-5	OOR <	OOR <		OOR <	OOR <	
IL-6	*1208 ± 120.1*	*940.5 ± 113.1*	ns	*842.7 ± 96.62*	*822.7 ± 128.6*	ns
IL-10	OOR <	OOR <		OOR <	OOR <	
IL-12p70	OOR <	OOR <		OOR <	OOR <	
IL-13	OOR <	OOR <		OOR <	OOR <	
IL-17	OOR <	OOR <		OOR <	OOR <	
G-CSF	*1977 ± 214.6*	*1769 ± 152.2*	ns	*845.2 ± 131.0*	*1488 ± 123.5*	**
GM-CSF	*25.21 ± 5.518*	*9.617 ± 2.116*	*	*41.16 ± 5.367*	*48.47 ± 9.601*	ns
IFN-γ	OOR <	OOR <		OOR <	OOR <	
CXCL1 (KC)	*2404 ± 451.6*	*3482 ± 741.9*	ns	*1877 ± 442.9*	*5045 ± 406.7*	***
CXCL2	*1476 ± 195.8*	*1676 ± 337.4*	*ns*	*1175 ± 243.9*	*2973 ± 141.2*	***
CXCL5	*619.5 ± 56.06*	*1085 ± 12.13*	***	*448.3 ± 34.48*	*1132 ± 21.11*	***
CXCL10 (IP10)	*195.0 ± 23.27*	*149.9 ± 16.86*	ns	*238.7 ± 59.80*	*179.0 ± 13.00*	ns
CCL2 (MCP-1)	*2259 ± 172.6*	*1983 ± 253.2*	ns	*2010 ± 102.3*	*2044 ± 88.66*	ns
CCL3 (MIP-1α)	*2017 ± 369.9*	*1546 ± 466.1*	ns	*1765 ± 272.9*	*1842 ± 227.5*	ns
CCL4 (MIP-1β)	*12586 ± 2099*	*11088 ± 3372*	ns	*10133 ± 986.2*	*10344 ± 866.9*	ns
CCL5 (RANTES)	*330.6 ± 86.79*	*171.1 ± 11.48*	ns	*377.8 ± 74.96*	*186.7 ± 16.34*	*
CCL11 (EOTAXIN)	*18.59 ± 4.075*	*18.56 ± 4.343*	ns	*9.499 ± 1.038*	*16.52 ± 2.249*	*
TNF-α	*184.1 ± 22.62*	*136.8 ± 27.07*	ns	*115.7 ± 14.86*	*138.9 ± 9.172*	ns
M-CSF	OOR <	OOR <		OOR <	OOR <	

Supplemental Table 6: cytokine/chemokine levels in BAL from *Rev-Erbα/β$^{fl/fl}$* and *Ccsp-Rev-Erbα-DBD^{m}/Rev-Erbβ$^{-/-}$* mice exposed to LPS at ZT0 or ZT12

Mice were exposed to aerosolised LPS (2mg/ml) at ZT0 or ZT12 for 20 minutes and culled 5 hours later. Cytokine/chemokine levels were measured using magnetic luminex assay. OOR< = Out of Range Below.

Values are presented as mean ± SEM, *n*=7-9, Student’s t test.

A

compound	nM	AhR	AR	ERa	FXR	GR	LXRa	LXRb	MR	NFkB	Nrf2
**reference**	Value at max fold activation	119	9.7	84	540	57	354	87	22	15	28
**DMSO**	0.10%	1.0	1.0	1.0	1.0	1.0	1.0	1.0	1.0	1.0	1.0
**1362**	10,000	4.6	1.4	2.8	1.7	1.7	1.8	1.3	1.3	1.3	2.6

compound	nM	PGR	PPARa	PPARd	PPARg	PXR	RARa	RXRa	TRa	TRb	VDR
**reference**	Value at max fold induction	114	25	179	37	31	5326	213	140	6518	7748
**DMSO**	0.10%	1.0	1.0	1.0	1.0	1.0	1.0	1.0	1.0	1.0	1.0
**1362**	10,000	1.2	1.1	1.1	3.6	8.6	0.31	1.8	0.21	1.3	0.77

B

compound	nM	CAR1	ERRa	ERRg	LRH-1	RORa	RORg
**reference**	Value at max fold induction	71	92	98	87	85	99
**DMSO**	0.10%	0.0	0.0	0.0	0.0	0.0	0.0
**1362**	10,000	-35	32	-75	-36	-23	5.9

C

compound	nM	AhR	AR	ERa	FXR	GR	LXRa	LXRb	MR	NFkB	Nrf2
**reference**	Value at max % inhibition	96	69	93	100	85	78	99	65	NA	NA
**DMSO**	0.10%	0.0	0.0	0.0	0.0	0.0	0.0	0.0	0.0	0.0	0.0
**1362**	10,000	-11	-46	-104	-25	-49	-41	-59	-98	13	-100

compound	nM	PGR	PPARa	PPARd	PPARg	PXR	RARa	RXRa	TRa	TRb	VDR
**reference**	Value at max % inhibition	98	NA	100	100	NA	99	100	NA	NA	NA
**DMSO**	0.10%	0.0	0.0	0.0	0.0	0.0	0.0	0.0	1.0	1.0	1.0
**1362**	10,000	23	-55	-75	-88	-41	-32	-67	13	-34	-0.96

Supplemental Table 7: Indigo screen data summary

(A) Summary of agonist assay results. Comparison of activation seen with GSK1362 compared to index agonist compounds. (B) Summary of inverse agonist assay results. GSK1362 did not exhibit inverse agonist activity (≥ 50 % inhibition) against any of the receptors tested. (C) Summary of antagonist assay results. NA indicates lack of commercially available, validated antagonist reference compound. GSK1362 did not exhibit antagonist activity (≥ 50 % inhibition) against any of the receptors tested.
